# Smartphone-Powered Automated Image Recognition Tool for Multianalyte Rapid Tests: Application to Infectious Diseases

**DOI:** 10.1021/acs.analchem.5c01487

**Published:** 2025-06-16

**Authors:** Marios Papadopoulos, Athanasios Kokkinis, Eleni Lamprou, Panagiota M. Kalligosfyri, Panagiotis N. Koustoumpardis, Despina P. Kalogianni

**Affiliations:** † Department of Chemistry, 37795 University of Patras, Rio, Patras GR26504, Greece; ‡ Robotics Group, Department of Mechanical Engineering and Aeronautics, 37795 University of Patras, Rio, Patras GR26504, Greece

## Abstract

Point-of-Care Testing (POCT) is rapidly increasing, providing quick, user-friendly, and portable diagnostic tools. Lateral flow assays (LFAs) have been central to POCT, administering fast and cost-effective diagnosis. However, traditional LFAs are limited to qualitative or semiquantitative results. The integration of artificial intelligence (AI) and image analysis with LFAs has significantly improved diagnostic accuracy, result automation, and quantification where applicable. ΑΙ/image analysis algorithms are trained to automatically correlate the visual results with the presence of the analyte in the sample. Smartphone-based devices increase accessibility but also face challenges such as strip positioning and background lighting, which image analysis can potentially address. This study demonstrates a smartphone and machine vision-driven multicolor LFA, as well as an additional independent AI tool, for detecting pathogens like and SARS-CoV-2 in a single test. The developed system was successfully applied to real samples, providing accurate and multiplex results, advancing the field of infectious disease diagnostics. The results are presented as color, text, and audio messages, meeting all special needs of the users.

## Graphical abstract

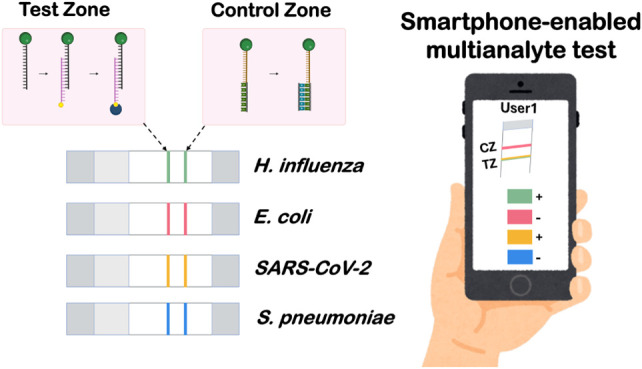

## Introduction

Point-of-Care Testing (POCT) is experiencing high growth for rapid diagnosis at the point of interest due to the advantages of Point-of-Care (POC) devices, including automation, user-friendly operation, and portability. Among the various POC technologies, lateral flow assays (LFAs), commonly referred to as “Rapid Tests”, have garnered significant attention from the research community. This interest has been particularly pronounced following their massive consumption and critical role during the COVID-19 pandemic. LFAs provide fast analysis, simplicity, cost-effectiveness, visual detection, portability, and disposability with applications in various fields, including biomolecular testing.^1^ In the case of infectious diseases, rapid and accurate diagnosis of the responsible pathogen plays a pivotal role in successful treatment. Rapid diagnosis is also crucial for controlling and preventing infection outbreaks, such as pandemics. LFAs have demonstrated substantial utility in healthcare applications.^2^

Nowadays, artificial intelligence (AI) and automated image processing have emerged in biomolecular analysis and rapid medical diagnostics as an attempt to increase the accuracy of testing, overcoming the need for extremely trained and qualified personnel, individuals, or the subjective interpretation of results that can lead to ambiguous conclusions. They have gained inconceivable attention from researchers worldwide. AI and image analysis tools have enhanced image-based diagnostics and disease diagnosis, increasing diagnostic accuracy, detailed interpretation, and analysis of complex medical images. They have also been beneficial to Analytical Sciences, from the design of innovative methods and nanomaterials to the development of sophisticated sensing platforms and “smart” technologies. AI/image analysis methods and algorithms have interrogated in developing (bio)­sensing technology, as they provide tools for better disease management and faster decision-making.^3,4^ AI and automated image processing exploit algorithms to uncover patterns and relationships and extract unique features from experimental data, enabling accurate detection, classification and categorization, and prediction of unknown samples. These tools enhance the capabilities of sensors in various scientific fields.^5^

LFAs provide rapid, sensitive, and specific detection of various biomolecules. Strip readers are available for the detection and quantification of LFAs. Colorimetric readers are composed of a broadband light source that illuminates the color lines of the strip; a sensor, usually a CCD camera or a CMOS sensor, for image acquisition; and a processor for image processing. However, the drawbacks of this technology are still false-negative and false-positive results, limitations in accuracy and multiple quantification, and portability issues.^6,7^ Many attempts have been made to increase the detectability and accuracy. Specific filters and specific wavelength selection for the illumination have been tried with the restriction that these strip readers can only be used with specific labels. Researchers have now turned to using advanced algorithms for image processing.^6^ On the other hand, smartphone-based POC devices, including LFAs, have further enhanced the simplicity and usefulness of the tests and overcame the need for special and expensive instrumentation, compared to strip readers. All the software needed is installed in mobile devices, while the great advantages of smartphones include the ease communication with healthcare facilities for real-time monitoring and the high flexibility.^6,8^ The analysis of LFA results using advanced AI and image processing tools has been shown to enhance diagnostic accuracy and facilitate quantitative assessment when required. To ensure correct interpretation in utilizing smartphone-based applications, these tools must address issues related to the orientation of the strips and background influences, such as environmental light. In addition, a current challenge in POC devices is the combination of biomarkers in a multiplex format so that clinicians can retrieve all the necessary information quickly for faster decisions.^4^ Communication of the results from POC devices to clinicians can also enable remote response.

Despite AI and image analysis advances, there are still limitations on their exploitation in LFAs. Therefore, there is a great need for these tools for POCT to enhance diagnostic efficiency beyond the traditional methods and to shorten the interpretation time.^1,2^ Regarding LFAs, all AI and/or image analysis-driven applications have been limited to single tests that provide response for one analyte only. These include the detection of HIV,^9^ SARS-CoV-2,^2,10,11^ IgG antibodies against SARS-CoV-2,^12^ CRP protein as a cardiac biomarker,^13^ hCG and cardiac troponin,^2^ high-density lipoprotein (HDL) and low-density lipoprotein (LDL),^4^ glycose,^14,15^ *Cryptococcal* antigen (fungi),^16,17^ methamphetamine and morphine,^18^ serum amyloid protein A,^19,20^ ractopamine and clenbuterol,^21^ spp,^22^ ,^23^ chorionic gonadotropin,^24^ ,^25^ drugs of abuse,^26^ and microRNA-21 or microRNA-96.^1^ There is only one report on a dual strip test with two test zones for the simultaneous detection of two analytes, namely IgG/IgM antibodies against SARS-CoV-2.^27^ Also, as reported in the literature,^28^ there are only two reports that exploit multicolor beads for the detection of three and four targets,^29,30^ respectively, but no image analysis or AI has been applied for the analysis of LFAs with colored beads as reporters.

This study demonstrates, for the first time, an AI, machine vision, and other image analysis tools-driven mobile (smartphone) interpretation of multianalyte/multicolor rapid tests—LFAs—for the simultaneous detection of up to four infectious diseases using colored beads as reporters. As models, we used single-stranded DNA (ssDNA) sequences that correspond to the 23S rRNA sequence of the pathogen bacteria , , and that causes the flu,^31^ as well as double-stranded DNA (dsDNA) sequences, obtained from real samples after PCR amplification, for the virus SARS-CoV-2 that causes COVID-19.^32^ Multicolor rapid tests that exploit beads of different colors as reporters were developed for distinguishing four different DNA targets. Beads of various colors—red, blue, green, and orange—were used for the detection of , , , and SARS-CoV-2, respectively, in a single rapid test. An automated system was also developed for the interpretation of test results with the objective of enabling the analysis of test strips targeting a range of infectious diseases. This system uses computer vision tools and mobile devices (e.g., smartphones) to determine whether the result was “Positive”, “Negative”, or “Invalid”. The most notable advancement of the integrated system lies in its robustness, as it operates independently of the strip’s orientation and background environment and remains unaffected by variations in ambient or technical lighting conditions. The developed system is color-agnostic, and no supportive accessories are needed, while previous systems depended mostly on fixed apparatus and predefined color codes. Toward this end, a mobile application (frontend) and a web server (backend) were developed, enabling any user to capture images of test strips and upload them to the server for subsequent analysis. The server communicates the results back to the user with the mobile application providing both visual and audio cues for accessibility reasons. Additionally, a web application has been created for users to access their test result history from a web browser and for users with elevated privileges (e.g., clinicians) to add or delete infectious diseases with different color inputs and handle all users’ results. Finally, an AI tool was developed as a separate tool but with a similar context to the image analysis tool’s context, exploiting the enhanced accuracy of AI for strip localization.

## Experimental Section

### Materials and Instrumentation

All reagents and instruments used in this study are presented in the Supplementary Information. All DNA sequences used are listed in Table S1.^31,32^

### Preparation of Colored Carboxylated Bead Conjugates (Reporters)

For the formation of the control zone of the strip and the detection of the SARS-CoV-2 virus, carboxylated beads conjugated with a dT(30) oligonucleotide probe were used. For the detection of the other three targets (3 bacteria), probes specific to the DNA targets were conjugated to the beads of different colors. All probes contained an NH_2_-group at 5′ end to enable conjugation. The conjugation reaction was performed as follows: An aliquot (10 μL) of colored beads (red, blue, green, and orange) was diluted in 100 μL of 0.1 M MES buffer, pH 4.5. After sonication for 2 min, the beads were collected by centrifugation for 10 min at 7000 g, resuspended in 200 μL of 0.1 M MES buffer, pH 4.5, and kept in suspension by another 2-min sonication step. Then, the carboxylate groups were activated by 10 μL of 10 mg/mL EDC prepared in 90% MES buffer and 10% H_2_O. The suspension was incubated for 15 min at room temperature, in the dark, with occasional stirring, and the addition of ΕDC was repeated using a freshly prepared solution. An amount of 100 pmol of each probe was finally added, and the mixture was left for conjugation for 60 min in the dark with occasional stirring. A volume of 2 μL of 10% (v/v) Tween-20 was added, and the beads were collected by centrifugation at 7000 *g* for 10 min. The beads were washed twice with 200 μL of 1×TE buffer (10 mM Tris-HCl and 1 mM EDTA at pH 8.0) and 2 μL of 10% (v/v) Tween-20. The beads were finally resuspended in 85 μL of 1× TE buffer and stored at 4°C.

### Amplification of SARS-CoV-2 by Polymerase Chain Reaction (PCR)

SARS-CoV-2 was amplified by PCR using a plasmid that contains a DNA sequence corresponding to SARS-CoV-2, as previously described.^32^ The amplified products were biotinylated using a biotinylated primer during PCR and analyzed with the multianalyte rapid test. The incorporation of a polydA sequence into the SARS-CoV-2 detection probe and the construction of the rapid test (LFA) are described in the Supplementary Information.

### Detection of the Targets by the Multianalyte/Multicolor Rapid Test—LFA

For the detection of ssDNA targets, 5 μL of each target at the appropriate concentration was hybridized with a mixture of the conjugated beads (5 μL each) for 10 min at 42°C. The hybrids were then deposited at the conjugate pad of the strip, and the strip was dipped into 350 μL of running buffer consisting of 1× PBS (pH 7.4), 0.1% (v/v) Triton, and 0.05% (w/v) SDS. The strip was left in the running buffer for 15 min. Finally, the strip was removed from the buffer and proceeded with visual detection of the targets and analysis by a mobile/web application.

For the detection of SARS-CoV-2 virus, a hybridization mixture was prepared by combining 1 μL of SARS-CoV-2 PCR product alone or in the presence of the other three DNA targets, 1 μL of a DNA probe specific to SARS-CoV-2 (1 pmol/μL), 1 μL of 900 mM NaCl, and 7 μL of 1× Kappa 2G Fast PCR buffer A, making a total volume of 10 μL. Next, the solution was mixed by vortexing and denatured at 95°C for 2 min in a thermocycler. After denaturation, the mixture was left for incubation at 42°C for 10 min to allow hybridization between the SARS-CoV-2 amplified sequence and the specific probe. After hybridization, we deposited 5 μL of the prehybridized mixture and 5 μL of dT(30)-beads onto the conjugate pad of the lateral flow strip. The strip was then immersed in 350 μL of the running buffer. The same procedure was followed as described above.

### Development of Mobile and Web Applications: *Backend of the System*

The backend part of the system oversees processing the user’s data (profile information, credentials, images). It is divided into a web server, a script for the test strip examination, and a database for the saved data. More specifically: (i) The Web server: A “NodeJS”, “ExpressJS”, application, which allows to easily add to the capabilities of the server. (ii) Script: For analyzing the photos. The OpenCV library in a Python (v 3.6.8) environment was used, since it contains ready-to-use functions. (iii) Database: A “MongoDB” database was used. Due to its “schemaless collections”, it allows the mutation of the data types that are being stored in it, which was proven to be useful for changes required during the development process. The web server handles and responds to user requests, while interfacing with the rest of the system through API end points with appropriate secrets and environment files. It also calls for the usage and piping of the data to the Python script that is in charge of images analysis. ***Front-end of the system.*** The front end of the system refers to the web and mobile applications and the elements with which the user can interact with. The mobile application was developed in the React Native framework. The user can register, login, view his disease history, see disease definitions, and send pictures for testing. The web application mostly serves the administrative users, allowing for the addition and deletion of disease definitions with “onClick­()”s and an html form using dynamic “color” inputs that returns a string of the hexadecimal notation (24-bit information, 3 8-bit channels). It was developed using Vanilla web development technologies and incorporates Bootstrap for styling and EJX for templating. It also features the Mobile application’s capabilities. The workflow of the web application is presented in Figures S1–S5.

### Analysis of the Multianalyte/Multicolor Strip Tests by the Developed Image Analysis-Driven System

The multianalyte test strips were analyzed and categorized using OpenCV (Open Computer Vision),^33^ an image analysis tool, and computer vision library, which enabled the fine control that allows the examination and manipulation of the uploaded images to the server. The most critical points behind the architecture of the program are as follows: (i) The Trailer class: a class that holds the information and methods that iterate through the length of the test strip and collect the data. (ii) The individual test strip extraction function: it is a function that uses global thresholding to extract the tests from the image into an array that holds the “sub-images”. (iii) Color range dictionary: whenever the program is called, the web server retrieves the color collection from the database and constructs a dictionary to iterate through it each time it finds a line to match a disease.

The workflow that is used by the script to determine the result of the strip is as follows: **1.** Test strip extraction from image: using Otsu’s binarization,^34,35^ the test strips are extracted from the image. Because Otsu’s method cannot be used to separate intersecting items, the test strips must not be in touch. **2.** Size filtering of contours: small contours are filtered out with OpenCV’s “contourArea”, to avoid small artifacts. **3.** Conversion of BGR to HSV color space: by applying color conversion (“cvtColor”) to gain the Hue, Saturation, and Value of each pixel, it will also be possible to determine the intensity and identity of the viral load. **4.** Image region extraction: the program analyzes each image region that contains a singular test strip for efficiency and because the current implementation requires the subimages to be horizontally aligned. As such, while the test strip orientation does not matter (Figure 5d), no strip should be touching another so that it can be correctly aligned. **5.** Horizontal alignment of strip subimages: by using the strip morphology and the salient orange part, the subimage is oriented horizontally, with the top orange part on the left. **6.** Iterate the pixels of the horizontal axis: the Trailer instance for each subimage creates an even, user-defined, number of points that iterate along the abscissa and calculates a mean for the H, S and V values of the pixels at each point. **7.** Local maxima detection: the “SciPy” function “findPeaks” finds the mean saturation local maxima indices and returns them to an array. “findPeaks” is applied after 30% of the length and before 80% to avoid spending resources on areas without lines and to avoid shadows. **8.** Categorize the type of result: the number, saturation value, and position of the maxima show whether the test is invalid, negative, or positive. The first line is the control line, which is also included in the disease color definition. **9.** Construct the color dictionary and match to a disease: using the “MongoPy” library, the disease data are pulled, and “sensitivity” is applied to the color to make hue ranges. The disease color array of the strip is then checked to see whether its colors fall into a range.

### Analysis of Samples by the Developed System

Finally, a series of samples were analyzed using the multicolor strip tests and the integrated machine vision-driven smartphone/web application. Samples containing different amounts of each target were hybridized to a mixture of the conjugated beads and analyzed by strip tests. Mixtures of two targets were also examined, and while there was success in this case, it is logical that its accuracy is bound by the quantization of the camera sensor and the beads used, and there may be color overlaps in specific case scenarios in future applications under certain conditions and with specific bead colors that are less salient. Images were captured by the smartphone and analyzed as described above by the mobile/web application. Results were visualized on the screen of the mobile device and were automatically recorded by the web application as an Excel file.

### Development of the Artificial Intelligence Tool

The proposed tool aims to resolve the limitations posed by the image analysis tool’s requirements, namely the need for a monochromatic background and the simplistic approach to decision-making regarding the test’s result. To that end, two separate neural network schemes were cascaded in a single flow to both segment the test strips and to apply a more sophisticated approach in the decision-making process. ***Segmentation network.*** The first part of the tool is the segmentation network. More specifically, it is an instance segmentation YOLOv8 model trained on strip tests without relying on environmental conditions. Multiple dataset augmentation techniques were used to ensure robustness in many different situations. Complementary technologies, like Slicing Assisted Hyper Inference (SAHI) were not used, because despite the number of pooling layers, the training and test images featured test strips of similar proportions to the image. Denoising and region-proposing algorithms were developed as assistive tools. ***Decision-making network.*** The decision-making network is a one-dimensional sliding-window convolutional neural network (CNN), aided by a similarly one-dimensional non-maximum suppression algorithm. Its fundamental principle of operation involves analyzing both the Saturation and Value/Brightness channels of the strips in the context of a Trailer object, similar to the image analysis tool, and it trained on a synthetic dataset that expanded upon the available dataset. In simple terms, it detects whether a region is a local maximum based on both channels, and the algorithmic logic determines whether it is invalid, negative, or positive based on the number of peaks and their relative positions on the test.

### Analysis of Samples by the Artificial Intelligence Tool

The tool itself follows the same flow as for the image analysis architecture. Many of the aspects of the previous tool were transferred to this iteration, most importantly the concept of the Trailer class and the logic behind the decision-making process, with some minor assistive algorithms regarding denoising through the morphological data of the candidates for detected instances of strips. The main difference is that it handles more efficiently a variety of different lighting and background conditions than the earlier system, which cannot process them successfully, resulting in a more fluid and realistic version of a system that could be used in an uncontrolled setting. While the image analysis tool provides more accurate results, it is unequivocal that the AI tool promotes practicality and applicability, enabling POC tests.

## Results and Discussion

In this study, we have developed new multianalyte rapid tests, LFAs integrated with a machine vision and image analysis and AI-driven smartphone-based detection system for the automated and accurate analysis of different infectious diseases. With the developed system, four different DNA targets could be distinguished in a single rapid test. The targets used as models are ssDNA targets that correspond to three harmful bacteria namely , , and , while the system was also applied for the detection of the virus SARS-CoV-2 after PCR amplification to count for real-sample application of the method. For the detection, different colored carboxylated microparticles—beads were conjugated to DNA probes specific to the four DNA targets and served as reporters in the multicolor rapid test. The color formed at the test zone indicated the target or the targets present in the sample. Red beads corresponded to , blue beads to , green beads to , and orange beads to SARS-CoV-2 virus. Targets were biotinylated at one end, and upon application to the strip, they were captured at the test zone by immobilized streptavidin and detected by specific DNA probes coupled to the colored beads. Beads conjugated to a dT(30) oligonucleotide were also used to form the control zone of the test through hybridization to immobilized polydA sequences (Figure 1).

**Figure 1:**
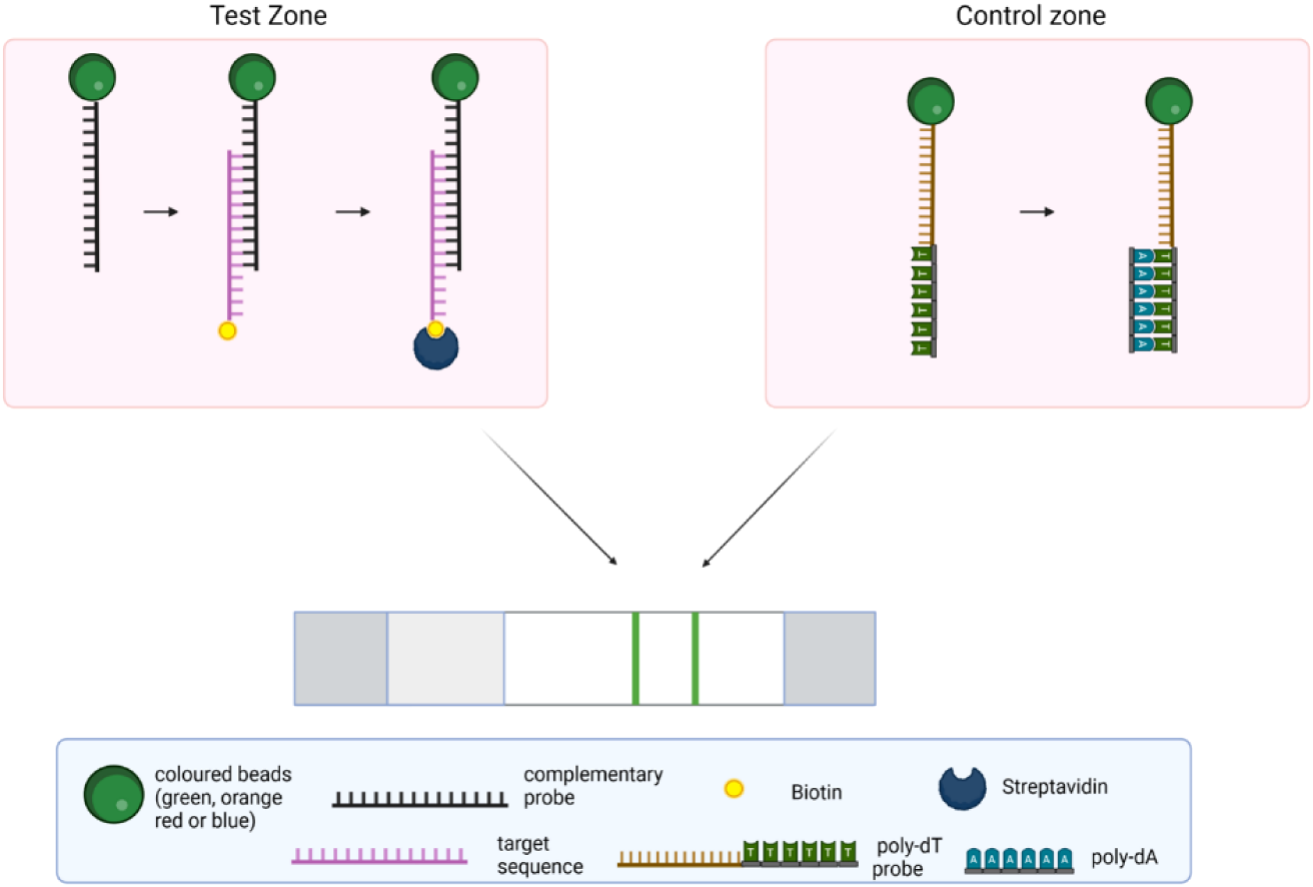
Principle of the multianalyte/multicolor rapid strip test. The targets are biotinylated and hybridized with a mixture of beads of different colors conjugated to the specific detection probes. SARS-CoV-2 detection probe is coupled to the beads through dA/dT hybridization using polydT-conjugated beads. The hybrids are captured by immobilized streptavidin at the strip’s test zone, forming a colored line depending on the target(s) present in the sample.

### Optimization Studies

Initially, optimization studies were performed in order to obtain the highest detectability and specificity of the strip tests. A series of valid and invalid strips of all four colors were prepared by spraying unconjugated beads onto the test and control lines of the strips using a 3D printer integrated with a technical pen as described by Kalligosfyri et al.^35^ (Figure S6). These strips served as the basis for both mobile and web applications. The types of strips used included valid strips containing both the test and the control zone and invalid strips that contained either the test zone or no zone.

The conjugation reaction was then optimized to obtain the most intense color at the test line of the strip with high specificity. All tests were performed with 100 fmol of a biotinylated-polydA (b-dA(30)) oligonucleotide as the target and dT(30) as the detection probe. Firstly, the amount of EDC was investigated. So different volumes (0.5–10 μL) of 100 g/L EDC diluted in H_2_O were tested with 10 μL giving the strongest signal (Figure 2a). Secondly, different amounts (10, 20, 50, 100, 200, and 400 pmol) of the detection probe were used, with 100 pmol giving the best result (Figure 2b). The running buffers for LFA were then examined. We chose the buffer that consisted of 1× PBS (pH 7.4), 0.1% (v/v) Triton, and 0.05% (w/v) SDS (buffer #7), which gave increased signal, better flow of the beads, less running time, and good clarity of the strip (Figure 2c). The reaction was also performed in the presence of sulfo-NHS for carboxyl group functionalization, but with no better result. Also, the resuspension of the conjugated beads at the last step with half-volume of the redispersion solution increased the signal (Figure S7a,b), as did the use of double the volume (20 μL) of the stock solution of the beads for the reaction (Figure 2d). Moreover, sonication of the beads, 5 and 10 s, between all steps of the conjugation reaction was tested, but no change was observed (Figure S7c).

**Figure 2:**
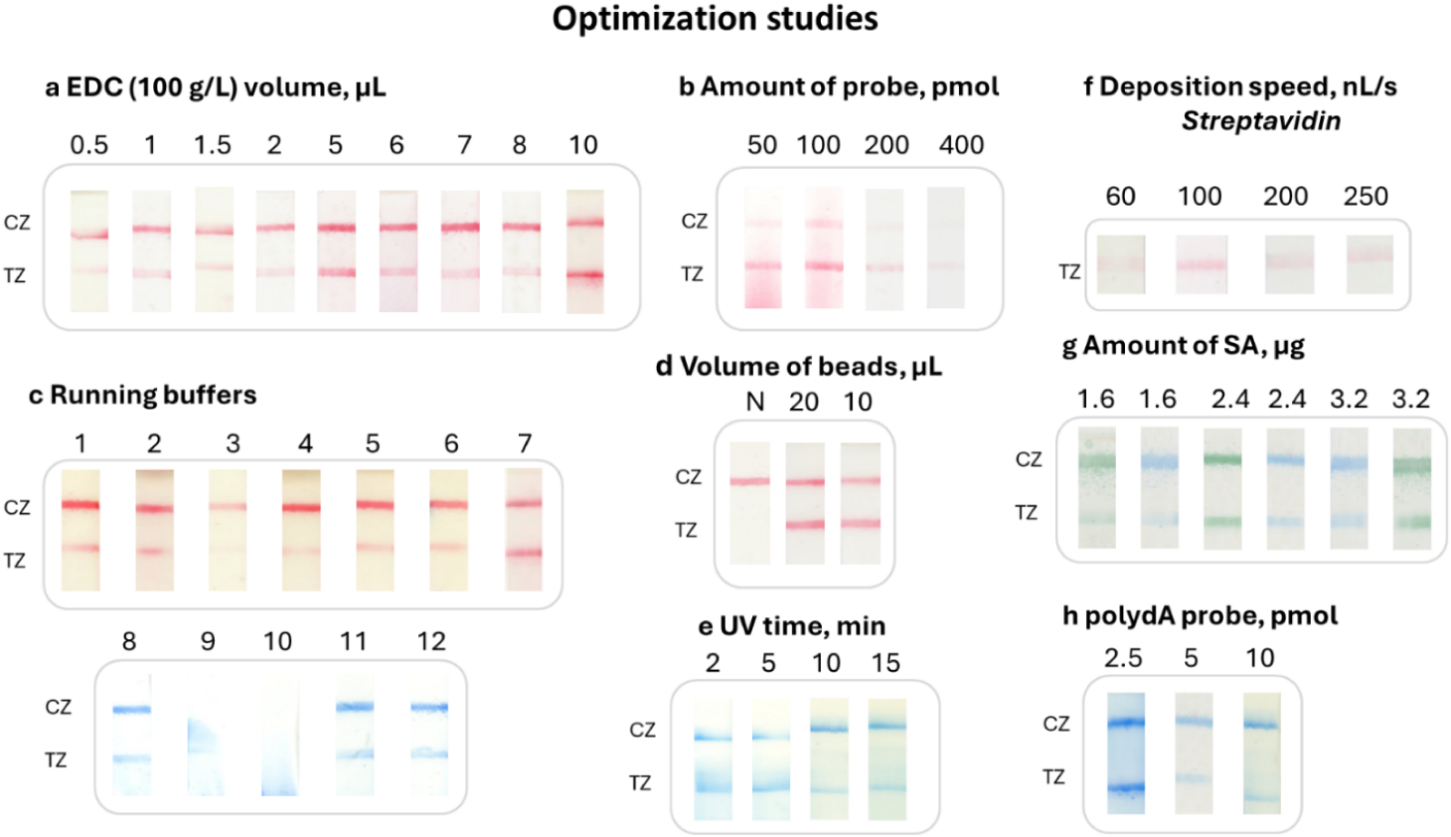
Optimization studies. *Conjugation reaction*. (a) Volume of EDC. (b) Amount of probe. (c) Composition of the running buffers. Different running buffers were tested in order to get a good flow of the beads and good clearness of the membrane of the strip with the most intense color (signal) at the test zone. 1: 2% Tween-20, 1× PBS pH 7.4; 2: 1× TE, 1% glycerol, 1% Tween-20; 3: 1% glycerol, 1% Triton X-100, 1× PBS pH 7.4; 4: 1% Tween-20, 1× PBS pH 7.4; 5: 2.5% Tween-20, 1× PBS pH 7.4; 6: 3% Tween-20, 1× PBS pH 7.4; 7: 0.1% Triton X-100, 0.05% SDS, 1× PBS pH 7.4; 8: 0.1% Tween-20, 0.05% SDS, 1× PBS pH 7.4; 9: 1× PBS pH 7.4; 10: 1% sucrose, 1× PBS pH 7.4; 11: 0.5% Triton X-100, 1× PBS pH 7.4; 12: 0.5% Tween-20, 0.5% sucrose, 1× PBS pH 7.4. (d) Volume of the stock of the beads. Deposition and immobilization of reagents on the membrane of the strip. (e) Time of UV irradiation. (f) Deposition speed for streptavidin. (g) Amount of immobilized streptavidin. (h) Amount of immobilized polydA probe. SA, streptavidin; N, negative; CZ, control zone; TZ, test zone.

Finally, the conditions for the deposition and immobilization of the reagents at the test and control zones of the strip were also optimized. For immobilization, the UV irradiation time was optimized at 5 min (Figure 2e). For the deposition of 2.4 μg of streptavidin (SA), different deposition speeds (60, 100, 200, and 250 nL/s) were tested using the Linomat 5 dispenser with 100 nL/s giving the most uniform line with the most intense color for both blue and green beads (Figure 2f). Finally, different amounts of SA (1.6, 2.4, and 3.2 μg) per strip and different amounts of polydA sequence (2.5, 5, and 10 pmol) were examined to form the test and the control line of the strip, respectively (Figure 2g,h). The amounts of 2.4 μg of SA and 2.5 pmol of polydA probe were selected to construct all strips for further experiments.

### Detectability of the Rapid Test

After optimization studies, the detectability of the multicolor rapid test was determined for all targets (colors). First, we tested the detectability of all beads of four different colors with the b-dA(30) probe, and then we analyzed different concentrations of all four targets of interest with the rapid test. In more detail, different amounts of the three bacterial targets (0–500 fmol) were used, while different DNA copies of SARS-CoV-2 DNA sequence (0–10^4^) were used in the amplification reaction and applied to the strip. As low as 6.25 fmol of b-dA(30) were detectable by the strips with all beads (Figure S8), while 1.56 fmol of and , 6.25 fmol of , and 10 copies of SARS-CoV-2 were detected (Figure 3). Furthermore, the detectability of the multicolor strip was also assessed in the presence of all of the beads during the hybridization reaction. As shown in Figure 3, the detectability of the strip was not influenced using the mixture of the beads, as 1.56 fmol of were also detected by the mixture with great specificity. Also, in the presence of two targets, as low as 3.1 fmol of and , as well as 6.25 fmol of and , were detected by the strip using all four beads with high specificity. For comparison, we have also constructed a calibration curve using a real-time polymerase chain reaction (PCR) and different DNA copies of the plasmid for SARS-CoV-2. As shown in Figure S9, as low as 100 copies were detectable by real-time PCR, while the strip detected as low as 10 copies, proving the very good detectability of the rapid strip test.

**Figure 3:**
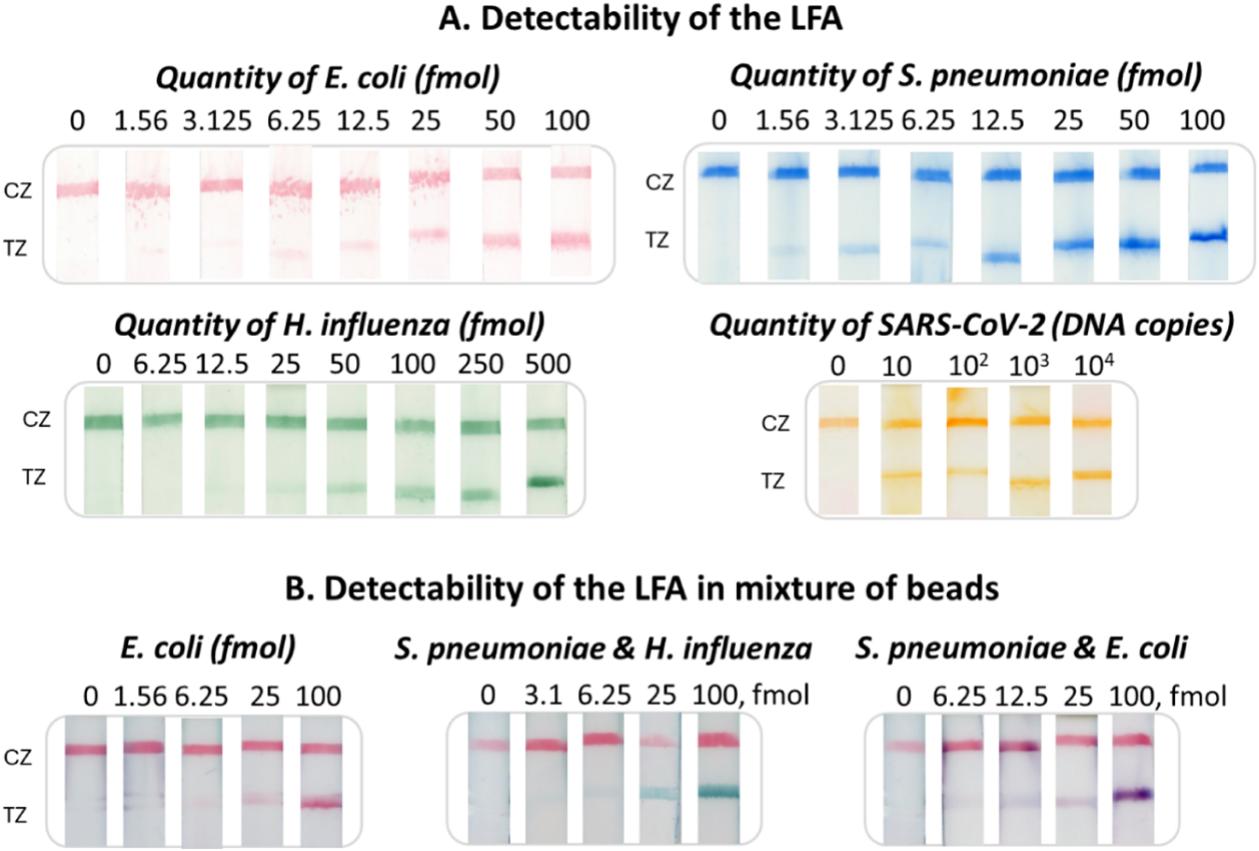
(A) Detectability of the rapid test for all four targets. Different amounts (0–100 or 500 fmol) were analyzed by strip test for each target and the corresponding colored beads. Detectability study for with red beads, for with blue beads, for with green beads, and for SARS-CoV-2 with orange beads. CZ, control zone; TZ, test zone. (B). Detectability of the rapid test in the presence of all beads. Detectability study for *E. coli* in a mixture of beads, for a mixture of and in the presence of all beads and finally, a mixture of and in the presence of all beads.

### Detection of the Targets Using a Mixture of the Four-Colored Conjugated Beads: Specificity of the Multianalyte Rapid Test

Furthermore, we evaluated the ability of the multicolor strip test to detect all targets by using a mixture of all four beads. In parallel, the specificity of the test for the four targets was studied. For the above tests, all targets (100 fmol) were analyzed in the presence of all four bead conjugates to test the specificity of the multicolor test. As observed in Figure 4a, only the color corresponding to the target present in the sample was formed at the test line of the strip, revealing the ability of the multicolor rapid test to distinguish all four targets, as well as the excellent specificity of the test. Then, mixtures of two targets in different amounts were prepared and analyzed with the multicolor test, as only one or two pathogens can simultaneously be present in the same organism. The results are also presented in Figure 4b. We observed the formation of test lines that displayed a combination of two colors depending on the targets used, as expected. By analyzing the strips with the developed application, the targets present in the mixture were successfully identified.

**Figure 4:**
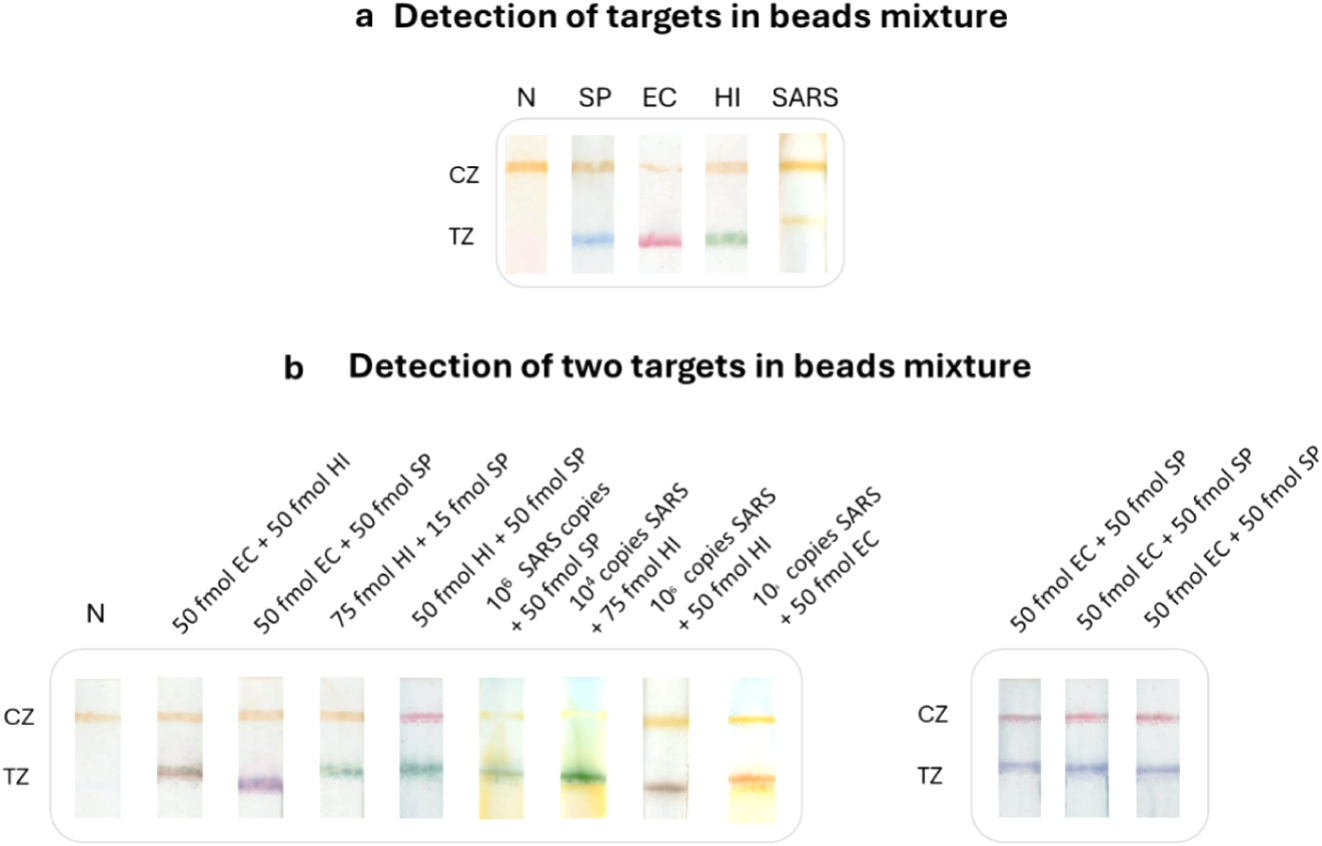
Detection of the targets in a mixture of the four beads and specificity of the multicolor test. (a) Each target was hybridized separately with the mixture of the beads. Only the color corresponding to the target present in the sample was formed at the strip’s test zone, proving the multicolor test’s excellent specificity. (b) Simultaneous detection of two targets with the beads’ mixture. N, negative; SP, ; EC, ; HI, ; CZ, control zone; TZ, test zone.

### Analysis of the Samples with the Developed Smartphone-Based Systems

Subsequently, a smartphone and web application were developed for the automated analysis of the strip tests of various samples for all four targets. The workflow of the user and the results of the mobile application for smartphones, tablets, etc., are presented in Figure 5. The user can register and login in to the application, where the history of the results is also available. The user can subsequently take a photo and upload it to the server where it is analyzed by the web application, and the results are communicated back to the mobile device. The result appears on the screen of the mobile device as a color and text message. An audio cue also comes along with the results to account for users with special needs. Multiple strip tests are deposited on a black background in any orientation. An image is finally captured and analyzed by the developed system.

**Figure 5:**
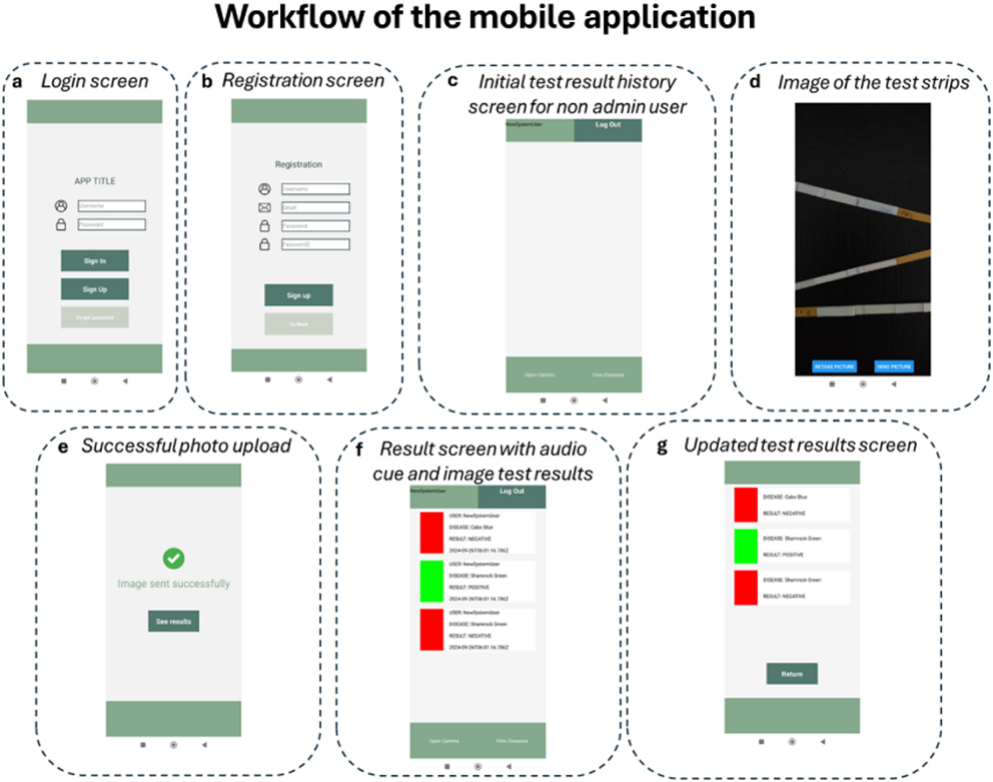
Workflow of the mobile application. (a) Login screen. (b) Registration screen. (c) Initial test result history screen for nonadmin user. The user must first log in or sign up with their credentials. Then, the test result page appeared on the screen. In this screen, the user can make one of the three following decisions: *(i)* Logout: it disconnects the user from the application and returns them to the login screen; *(ii)* Navigate to the disease screen that displays defined by its name and the color of the test line of the strip, which categorizes the strip as positive. (iii) Navigate to the camera screen: (i) Green, which means positive to the disease; (ii) Red, which means negative to the disease; and (iii) Yellow, which indicates the infectious diseases identified by the system. An infectious disease is characterized by its name and the color of the test line of the strip that categorizes the strip as positive and *(iii)* Navigate to the camera screen: navigate to the camera screen to take a picture of the test strips. (d) Image capture of the test strips. (e) Successful photo upload. (f) Result screen with audio cue and image test results. (g) Updated test results screen. After taking a picture, the user will receive a confirmation message on the screen indicating that the image was sent successfully. If the test results are positive for an infectious disease, an audio cue will be played, and a screen will display a vertical list of the diseases for which the user has tested positive for. Finally, the user is directed to the test result history screen, which displays the updated information.

As for the distinction of the four colors formed at the test line of the strips by the developed application, the results of the analyzed bead mixtures have shown that the different colors were effectively distinguished. The different colors were efficiently distinct in the HSV (Hue, Saturation, Value) color space without any overlap in their ranges. In more detail, one value is saved for each color, which is then translated to a range for that specific color. The algorithm itself works in the following way: *(1)* The colors are read from the database, and the ranges are calculated. *(2)* The local maxima for the Saturation values are found using the find_peaks­() scipy python function. The number and positions of the local maxima are the data that determine the results of the test itself. *(3)* The Hue value is read, and the specific analyte(s) (infectious disease) in the samples is determined. This approach is adequate for the four different colors of the beads chosen, as they are spread enough in the Hue color wheel.

For the validation of the proposed method, we subsequently determined the accuracy, clinical sensitivity, and specificity of the system by analyzing a series of various samples. Samples that contained different amounts of targets were prepared, hybridized with a mixture of the four bead conjugates, and analyzed using the multianalyte rapid test and the developed application. A total of 185 samples were analyzed. The strips from the analysis of all positive samples and representative negative samples are presented in Figures S10–S13. In addition, for the validation of the AI tool, a total of 120 images of strip tests containing different amounts of the targets, as well as nasopharyngeal samples for SARS-CoV-2, were analyzed.

The interpretation of the results for both image processing tools is provided using a confusion matrix analysis (Figure 6). An excel spreadsheet was used to record both the actual and predicted results of a binary classification task, with “1” representing positive samples and “0” representing negative samples. The actual results were obtained with visual inspection, while the predicted results were generated by the machine vision application. The “COUNTIF­()” function in Excel was utilized in order to compute the four fundamental outcomes for the confusion matrix: a) True Negative (TN): Actual value = 0 and Predicted value = 0, b) False Positive (FP): Actual value = 0 and Predicted value = 1, c) False Negative (FN): Actual value = 1 and Predicted value = 0, d) True Positive (TP): Actual value = 1 and Predicted value = 1. All negative samples were correctly classified as negative. For the image analysis tool, a total of 126 out of 130 positive samples were correctly identified, while 4 were misclassified as negative (false negatives). By applying Excel formulas, we determined the accuracy, sensitivity, and specificity of the application, which resulted in values of 97.8%, 97.0%, and 100% respectively, proving the very good performance of the developed multicolor strip test integrated with the machine vision and image analysis smartphone-based application. The system was also applied for the analysis of mixtures of two targets with the strip tests presented in Figure 4. All samples were correctly classified for the target present in each sample. For the validation of the AI tool, (i) a total of 165 images of test strips were analyzed, 157 of which (95.1%) were correctly localized in the images that contained 1–3 strips and (ii) a total of 120 strip tests were analyzed, out of which all negative samples were correctly found as negative, while 73 of the 89 positive samples were correctly classified as positives, and 16 samples were false negatives. Therefore, the AI tool provided 86.7% of accuracy, 82.0% of sensitivity, and 100% specificity. Based on the results, the AI tool was more useful for enhancing the accuracy of strip localization in a photo under various environmental conditions. Finally, nasopharyngeal samples obtained from volunteers that were found negative or positive for COVID-19 were subjected to analysis with the multicolor strip test and with real-time PCR against SARS-CoV-2 for comparison. The results are presented in Figure S15. The two methods showed very good agreement, proving the very good potential of the proposed method as a diagnostic tool.

**Figure 6:**
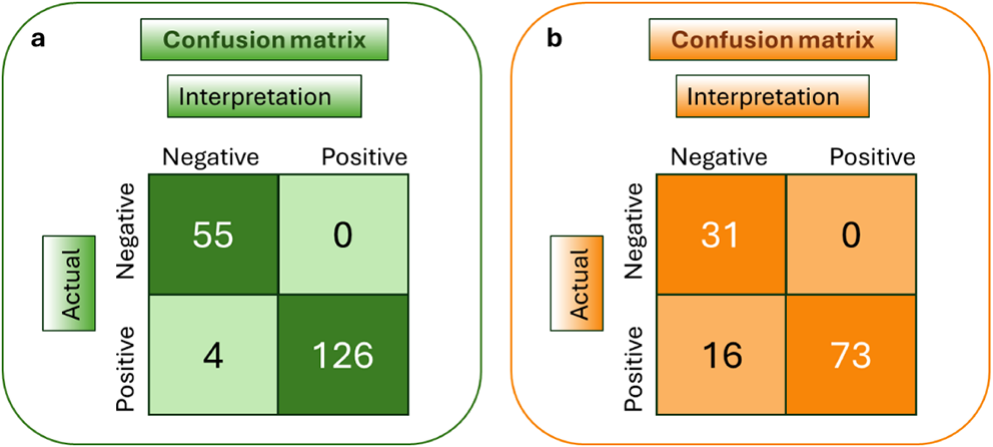
Confusion matrix. The actual classification of the samples was compared to that of the interpretation of the visual outcomes of the strip tests of all analyzed samples by the developed systems. (a) Image analysis-based system and (b) AI-based system.

### Repeatability of the Multianalyte/Multicolor Rapid Test

Firstly, the repeatability of the performance of the multicolor rapid strip test was determined by analyzing in triplicate 25, 50, and 100 fmol of the b-dA(30) probe (Figure S14). The strips were analyzed by the free online ImageJ software. After densitometric analysis of the test zones of the strips, we calculated the coefficients of variation (%CVs), which were 1.1%, 1.1%, and 0.3% for 25, 50, and 100 fmol, respectively, proving the excellent repeatability of the multicolor. To ensure the repeatability of the multicolor test, most samples of all targets were analyzed in triplicates. As observed in Figures S10–S12, all strips with all four-colored beads demonstrated very good repeatability. The results for the repeatability of the application for the analysis in triplicate of representative samples are presented in Table S2, while the images of the strips are presented in Figure S16. All triplicates were correctly classified. Moreover, an amount of 100 fmol of , , and was analyzed four, six, and seven times, respectively. The %CVs were found to be 7.1% for , 8.9% for , and 6.8% for , all showing the great repeatability of the assay. We also determined the repeatability of the performance of the multicolor test in mixtures of two targets. A mixture of and (50 fmol of each target) was analyzed in triplicate (Figure 4b). The %CV was 2.1%, proving the very good repeatability of the test in the mixtures. Finally, the AI-based application was used to assess the intra- and interdevice repeatability. For intra-device repeatability, a series of samples containing one or two analytes were analyzed with the multicolor strip test, and several photos of every strip were obtained using the same smartphone to assess the intrarepeatability or different mobile devices for inter-device repeatability determination. The results are presented in Table S3 and proved the very good intra- and interdevice repeatability of the smartphone-based system.

## Conclusions

We have developed a multicolor rapid test integrated with a smartphone-based application along with image analysis and machine vision tools for the multianalyte detection of infectious diseases. This is the first time that such an automated system has been developed for the “reading” and interpretation of multiplex rapid strip tests capable of detecting up to four analytes using colored beads as reporters. Each colored line formed on the strip represented a specific pathogen present in the sample. A key point of this research was the successful detection of mixed infections, where different color combinations were formulated at the test line, suggesting the presence of different pathogens and enabling the simultaneous identification of multiple infections. The combination of multiple-colored beads in a single line compared to four different test lines enabled the construction of a universal strip that can be used for any other analytes by simply changing the detection DNA probes attached to the surface of the beads.

The system application consists of a mobile (smartphone) and a web application for personal use or use by professionals (e.g., medical personnel). The mobile application can examine multiple test strips at once (up to 10) and in any direction and under environmental conditions, avoiding the need for multiple pictures of single tests. Furthermore, the audio cues, colors, and text messages help users of every capability and special needs with the interpretation of the results, ensuring that every user’s need is met as efficiently as possible. Another advantage of the system is that, by using the “expo-camera” package, the “auto white-balancing” feature is activated, applying color correction, and enabling the user to take pictures under different light conditions. The main disadvantage of the system, however, is that it requires a solid and contrasting background (black), as the machine vision script applies a global threshold to extract the tests from the background. It is important to note that although the system is capable of analyzing multiple test strips positioned at varying angles and orientations, the strips must be placed separately without physical contact to ensure an accurate interpretation. After analyzing a series of samples, the system showed very good accuracy (97,8 %), sensitivity (97%), and specificity (100%). Moreover, when two targets were simultaneously present in a single sample, the system successfully identified the targets. The additional tool is another developed solution that addresses the issues of the first application by eliminating all of the requirements for stable operations. While it supports the necessary operations, it could benefit from testing of different instance segmentation and CNN architectures. The highlight of this tool is the fact that it could detect and separate the strips more reliably while also using a more generalized approach in translating the colored values into a meaningful test result.

This research suggests immense potential for its future development. Some applications extend toward comprehensive diagnostic platforms in fields such as personalized medicine and epidemiological monitoring. This technology could also be applied to identify bacterial coinfections. The function of remote detection and its portability ensure that even in underfunded healthcare systems, rapid diagnostics could be feasible, empowering healthcare providers for faster clinical decisions. Most importantly, this low-cost and easy-to-use platform transcends traditional POCT by offering improved accuracy, multiplexing capabilities, and real-time decision-making, ensuring that even the most remote communities can access advanced healthcare solutions. The developed machine vision-driven automated mobile/web system is highly flexible and could be exploited in any rapid test with any reporters. In the future, in combination with an easy immunoassay-based strip test, the audio and color cues provided by the application would enable their use, even by people with special needs. However, more effort is required for further development of the system to enable the quantification of multiple targets in a sample. LFAs offer a more cost-effective alternative to real-time PCR, primarily because they do not require expensive or specialized instrumentation. In contrast, real-time PCR designed for multiplex detection necessitates advanced and costly equipment equipped with multiple detection channels. Furthermore, the multiplexing capability of real-time PCR is limited due to the spectral overlap of the fluorescent dyes employed as labels, which restricts the number of analytes that can be simultaneously detected. In this context, LFAs exhibit superior potential for multiplexing. The multiplicity of LFA can be further enhanced by incorporating new reporters of different colors. In conclusion, the proposed system, which integrates smartphone-based technology with machine vision and automated image processing, represents a significant advancement in the field of rapid diagnostic testing of infectious diseases. Moreover, the AI tool shows that it is possible to integrate a solution to the earlier system and combine it with the mobile application, as it is now a desktop-only realized tool. Furthermore, the test strip format and its applications are highly versatile, allowing for adaptation to a wide range of target analytes. This means that the proposed system can be easily adapted for other infectious diseases by simply changing the recognition DNA probes, increasing the general applicability of the strip test.

## Supplementary Material



## Notes

The materials, solutions, and instrumentation; the nucleotide sequences used in the study; detailed information about the development of mobile and web application; further optimization studies; the detectability study with ssDNA; and the analysis of a series of samples of all four targets (PDF)
