# Supplementary material for: Smartphone-Powered Automated Image Recognition Tool for Multianalyte Rapid Tests: Application to Infectious Diseases
Source: Anal Chem. 2025 Jun 16;97(25):13340–9. doi: 10.1021/acs.analchem.5c01487 (PMC12224150; doi:10.1021/acs.analchem.5c01487)
Supplement: Supplementary file 1 [file ac5c01487_si_001.pdf]

Supporting Information: Smartphone-Powered Automated Image Recognition Tool for Multianalyte Rapid Tests: Application to Infectious Diseases

Marios Papadopoulos[1,#], Athanasios Kokkinis[2,#], Eleni Lamprou[1,#], Panagiota M. Kalligosfyri[1,*], Panagiotis N. Koustoumpardis[2] and Despina P. Kalogianni[1,*]

[1]Department of Chemistry, University of Patras, GR26504, Rio, Patras, Greece.

[2] Robotics Group, Department of Mechanical Engineering and Aeronautics, University of Patras, GR26504, Rio, Patras, Greece.

[#] Equal contribution

* Corresponding authors

Despina P. Kalogianni, kalogian@upatras.gr

Panagiota M. Kalligosfyri, pkalligosfyri@gmail.com

Table of Contents

Contents	Figures & Tables	Pages
Materials and Instrumentation		S3
The sequences of DNA targets and DNA probes	Table S1	S4
Solutions		S4
Incorporation of a polydA sequence to the SARS-CoV-2 detection probe		S4
Construction of the rapid test – LFA		S5
Development of mobile and web application		S5
Web server - Requests and basic routing		S5
Script and Libraries		S5
Database middleware		S6
SciPy		S6
User information scope		S7
Mobile application – Expo		S7
Web application		S7
Web Application login page	Figure S1	S8
Web application registration page	Figure S2	S8
Example of the result history page for the administrator user	Figure S3	S9
Web page for an administrator to input or delete a color input	Figure S4	S9
Example of adding/inputting a color input	Figure S5	S9
Results		S10
Examples of the strips used for the development of the mobile and web applications – 3D printed zones	Figure S6	S10
Optimization studies of the coupling reaction	Figure S7	S10
Detectability of the strip test for ssDNA	Figure S8	S11
Real-time PCR protocol for SARS-CoV-2		S11
Calibration graph for SARS-CoV-2 with real-time PCR	Figure S9	S12
Analysis of *E. coli* samples with the strip test	Figure S10	S13
Analysis of *S. pneumoniae* samples with the strip test	Figure S11	S14
Analysis of *H. influenza* samples with the strip test	Figure S12	S15
Analysis of SARS-CoV-2 samples with the strip test	Figure S13	S16
Repeatability of the multicolor strip test	Figure S14	S16
Analysis of real samples for SARS-CoV-2	Figure S15	S17
Repeatability of image analysis-based application	Table S2	S18
Intra- and inter-repeatability of the AI and image analysis-based application	Table S3	S19

Materials and Instrumentation

Materials

The red and orange carboxylated microspheres had a diameter of 0.297 μm and 0.21 μm, respectively and were purchased from Polysciences Europe GmbH (Hirschberg, Germany), while the blue and green carboxylated beads of 0.197-μm and 0.196-μm diameter, respectively, were from Bangs Laboratories, Inc (Fishers, Indiana,USA). The terminal transferase (TdT) was from New England Biolabs (Ipswich, Massachusetts, USA), the 2-(N-morpholino)ethanesulfonic acid (MES) from Sigma-Aldrich (Saint Louis, Missouri, USA), the 1-ethyl-3-(3-dimethylaminopropyl) carbodiimide (EDC) from Apollo Scientific (Chesire, UK) and streptavidin was purchased from Roche (Basel, Switzerland). The PrimeScript 1st strand cDNA synthesis kit was form TaKaRa (Kusatsu, Japan) and the Kapa2G Fast PCR kit (Kapa Biosystems, Basel, Switzerland) was used for Polymerase Chain Reaction (PCR). The synthetic DNA oligonucleotides were from Eurofins Genomics (Ebersberg, Germany) (Table S1). The nitrocellulose membrane FF80HP, the Standard 17 glass fiber conjugate pad and the blotting paper Grade GB003 used as immersion and absorption pad were from Cytiva (Marlborough, MA, USA). All the other common reagents were purchased from Sigma-Aldrich (Saint Louis, Missouri, USA).

Instrumentation

PCR and hybridization reactions were performed in the PCR Thermal Cycler Dice TP60 (Takara Bio, Kusatsu, Japan). The construction of the test and the control zone of the rapid tests was performed using the CAMAG Linomat 5 (Muttenz, Switzerland) and the UVP Crosslinker CL-3000 (Analytik Jena, Germany). The images of the rapid strip tests were obtained with the scanner EPSON Perfection V500 PHOTO (Seiko Epson Corporation, Suwa, Japan). For the conjugation of beads, the ultrasonic bath used was the RK52H (Bandelin electronics, Berlin, Germany) and the centrifuge MIKRO 185 (Hettich, Tuttlingen Germany). Finally, during experimental processes the mini centrifuge NG002B (NIPPON Genetics, Duren, Germany) and the Vortex V-1 Plus (Kisker Biotech, Steinfurt, Germany) were used.

Table S1 The sequences of DNA targets and detection DNA probes used in this study[31,32]

Oligonucleotide Name	Sequence 5'→ 3'
DNA targets	
Escherichia coli	Biotin-TTCCACTAACACACACACTGATTCAGGCTCTGGGCTGCT
Streptococcus Pneumioniae	Biotin-TAATCTTTGAGTCCACATTGCAGTCCTACAACCCCGAAGA
Haemophilus Influenza	Biotin-TTTGATTGTGCTTCCCAACACATTCTCCTCACTATATTGC
DNA Probes	
E. coli complementary probe	NH_2-CCAGAGCCTAATCAGTGTGT
S. Pneumioniae complementary probe	NH_2- GGTTGTAGGACTGCAATGTGGACTC
H. Influenza complementary probe	NH_2-GTGAGGAGAATGTGTTGGGAAG
SARS-CoV-2 detection probe	ACCCCGCATTACGTTTGGTGGACC

Solutions

The 6×Saline-Sodium Citrate (SSC) consisted of 0.9 M NaCl and 90 mM sodium Citrate, adjusted to pH 7.0. The 1×Phosphate-buffered saline (PBS) solution consisted of 137 mM NaCl, 2.7 mM KCl, 8 mM Na_2HPO_4 and 2 mM KH_2PO_4, adjusted to pH 7.4.

Incorporation of a polydA sequence to the SARS-CoV-2 detection probe. For the detection of SARS-CoV-2, a polydA sequence was incorporated into the detection probe by the enzyme terminal deoxynucleotidyl transferase (TdT) to enable conjugation and detection with the rapid test. The reaction (20 μL) contained 1×TdT buffer, 0.25 mM $CoCl_2$, 2 mM dATP, 400 pmol of the probe and 30 U of the enzyme TdT, and was performed at 37 °C for 1 h, followed by the addition of 2 μL of 0.5 M EDTA at pH 8.0 to inactivate the enzyme and pause the reaction.

Construction of the rapid test – LFA. The rapid test – LFA consisted of four parts: an absorption pad, a nitrocellulose membrane, a conjugate pad, and an immersion pad, assembled on a plastic adhesive pad with a width of 4 mm and a height of 70 mm. The construction of the test and the control zone on the diagnostic membrane of the strip was implemented by immobilization of 2.4 μg streptavidin (SA) and 5 pmol of a polydA sequence, respectively. For the deposition of the reagents, SA was prepared at a concentration of 2.4 μg/μL and the polydA sequence 5 pmol/μL in 5% (v/v) MeOH, 2% (w/v) sucrose and 6×SSC pH 7.0, and deposited onto the membrane using the automated dispenser Linomat 5 (Camag, Switzerland) at a speed of 100 nL/s. The immobilization was finalized in the UV crosslinker CL-3000 (Analytik Jena, Germany) by applying an energy of 125 mJ/cm^2 for 5 min. All the parts of the strip were properly assembled onto an adhesive pad with overlapping ends to result in a continuous flow along the strip.

Development of mobile and web applications

Web server - Requests and basic routing

The routing of the application and the way the server interacts with the users differ from what has already been reported in the literature. It is a standard Express web server, without obfuscation or hardening with complementary packages (e.g., “Helmet.js”), exploiting the feasibility of using mobile devices to determine the result of the tests. For the users, hashing and salting have been used for the filtered passwords (protection of data on rest) alongside the SSL protocol for the requests (protection of data on transit). This ensured that the minimum-security requirements were met. The server is also responsible for saving the uploaded images for future reference. This is done using the “FileSystem” package. Regarding the technical requirements for the server to work, as well as the rest of the system, is to configure the Dynamic Host Configuration Protocol (DHCP) server of the network to bind the Media Access Control (MAC) address of the host that is hosting the server to gain a static local Internet Protocol (IP) address for the host.

Script and Libraries

The script was developed in the Python programming language (version 3.6.8). The main libraries used in this project were OpenCV, MongoPy, and SciPy. The “PythonShell” package was elected as the optimal way to call the script through the Express server. This approach is less resource

intensive than building another server, even something as lightweight as a Flask server that responds to requests from the Express application. The image is passed through the system arguments during the program's call from the Express application.

Database middleware

It is common practice to use the "Mongoose.js" middleware to communicate with a MongoDB database. The use of Mongoose has made it possible to use the prototypes given by the package to make and alter "Schemas", which are the objects' structures that are saved in the MongoDB database. Simultaneously, it is possible to define how they behave during the writing process of their instances on the database. For example, in the Schemas, the hashing and salting processes are defined to be used later during user creation. The Schemas are then used to make "Models" that are being used to interface, query and filter results from the "Collection" (table) that each Model defines. The Schemas that were created are:

- User: this Schema holds the user's username, email, hash digest of the password and whether the user is an admin or not.
- Infectious Disease: this Schema contains the name of the disease and an array of colors representing it.
- Result: this Schema holds the disease's name, the user who sent the image, the result, as well as the date and time it was uploaded.

The local database ("on-premises database") itself can be downloaded for free, with the organization that made MongoDB offering a paid cloud storage solution with free tiers ("MongoDB Atlas"). As such, not many configurations had to be made, as the database was downloaded locally and without the need for port-forwarding. For the elevation of the user privileges to admin users, we chose the "Mongo Compass" tool for the direct flip of the Boolean data field of the user schema to "true" for the "admin" value.

SciPy

SciPy (Scientific Python) is a package that contains many different modules for data analysis. For this research, it was used to find the local maxima of the mean HSV values of the sub-images with the "findPeaks" function. The parameters for this function were empirically determined after many

tests that were not included in the results section and were only used for validation and development purposes.

User information scope

Each user should only be privy of the information that concerns them and no other users of the system. That much can be immediately understood by thinking how someone reading someone else's medical information would be construed as intrusive. Even then, there should be users who can see every user's test history. These users may be medical personnel or even lead researchers in a lab.

For the sake of following conventional practices, the users are classified as "admin"s if they are to be able to see everyone's test results, and "non admin"s if they should only be able to see their own test results. Additionally, admins can add and delete diseases to and from the system from the web application. In contrast, users without elevated privileges can only view the diseases in both the web and mobile applications. It is stressed that the admins will not be able to input a disease that already exists in the Collection, ensuring that no duplicate names will be present.

Mobile application – Expo

"Expo" is a framework built on the React Native framework that further simplifies the creation of mobile applications, with extensive functions that work effortlessly with the most popular operating systems. More specifically, it was used for navigation, camera usage, and user permission request parts. Expo also allowed for the application to be built on the cloud server that is offered with the free plan, as well as the platform known as "Expo Go" which is a mobile application and SDK (Software Development Kit) that assists with the testing of the application on physical mobile devices. For compatibility reasons, version 49.X.X of the SDK was used for Android devices.

Web application

Web application has been developed to be used by administrators. Some typical examples of the application's environment are presented in Figures S1-S5.

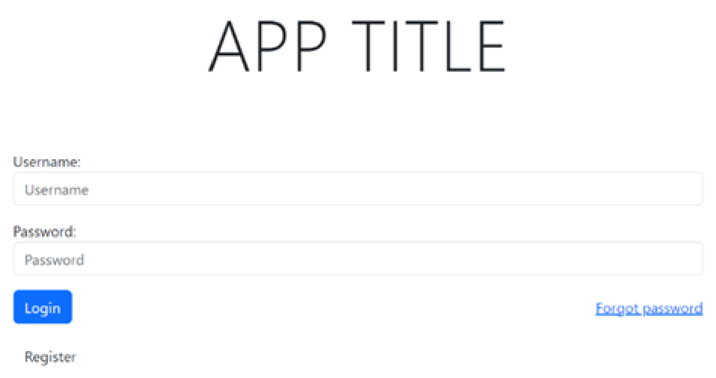

Fig. S1. Web application login page. App: application.

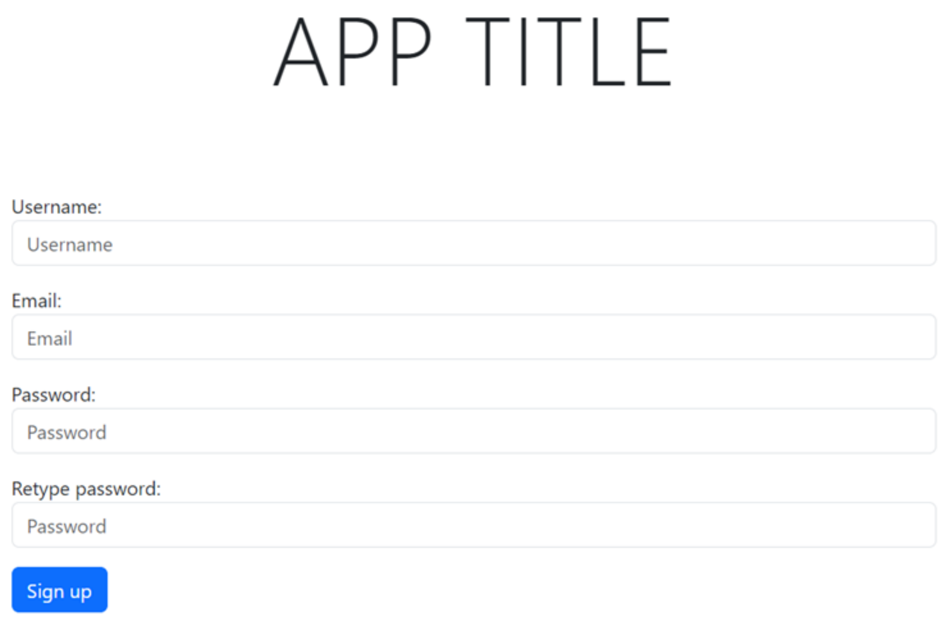

Fig. S2. Web application registration page.

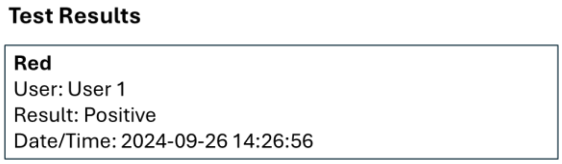

Fig. S3. Example of the result history page for the administrator user.

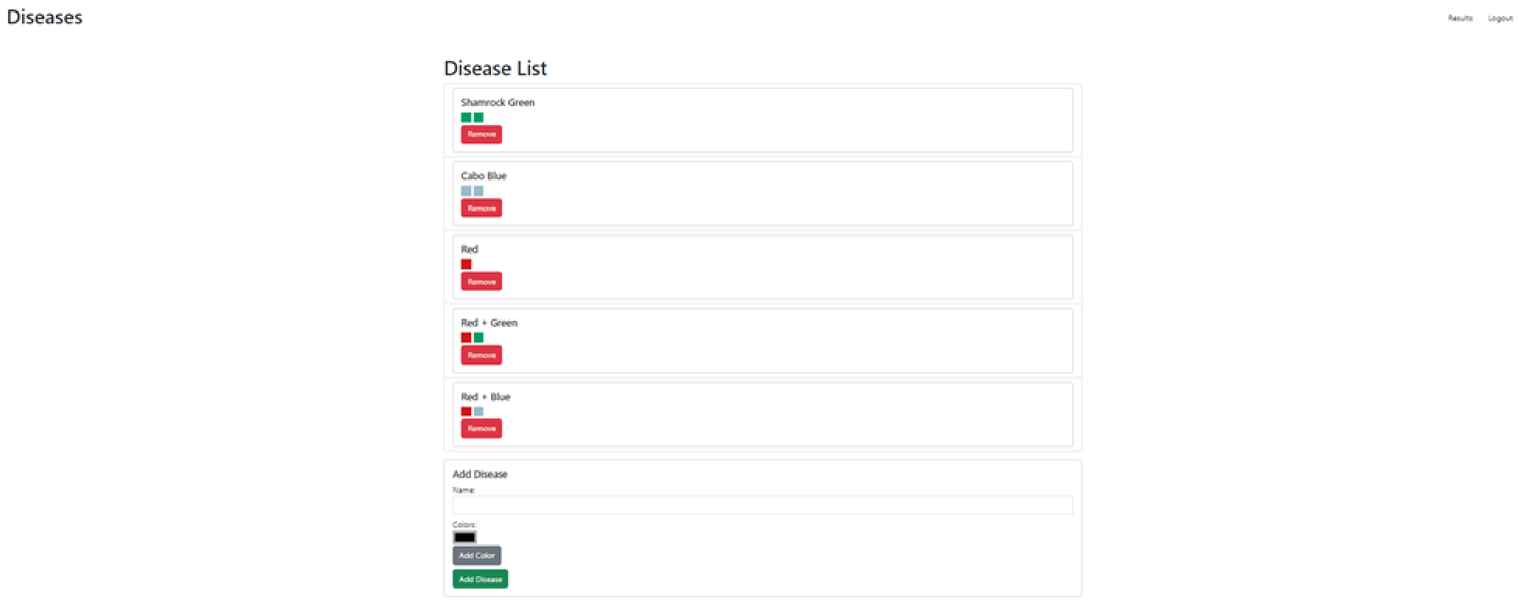

Fig. S4. Web page for an administrator to input or delete a color input, thus an infectious disease.

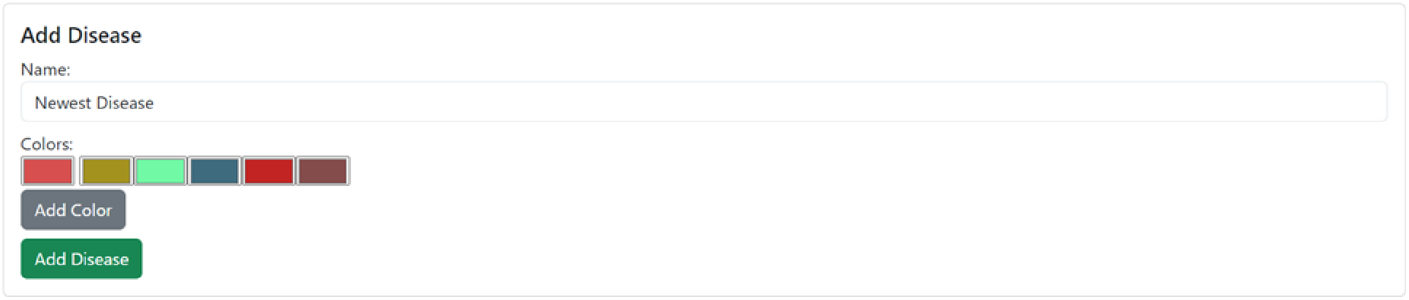

Fig. S5. Example of adding/inputting a color input, thus an infectious disease.

Results

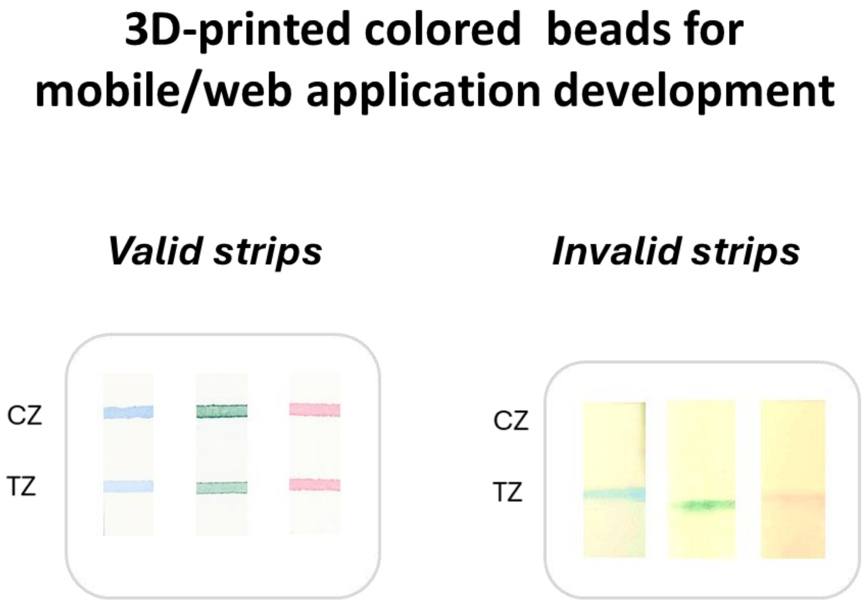

Fig. S6. Examples of the strips used for the development of the mobile and web applications. CZ: control zone, TZ: test zone.

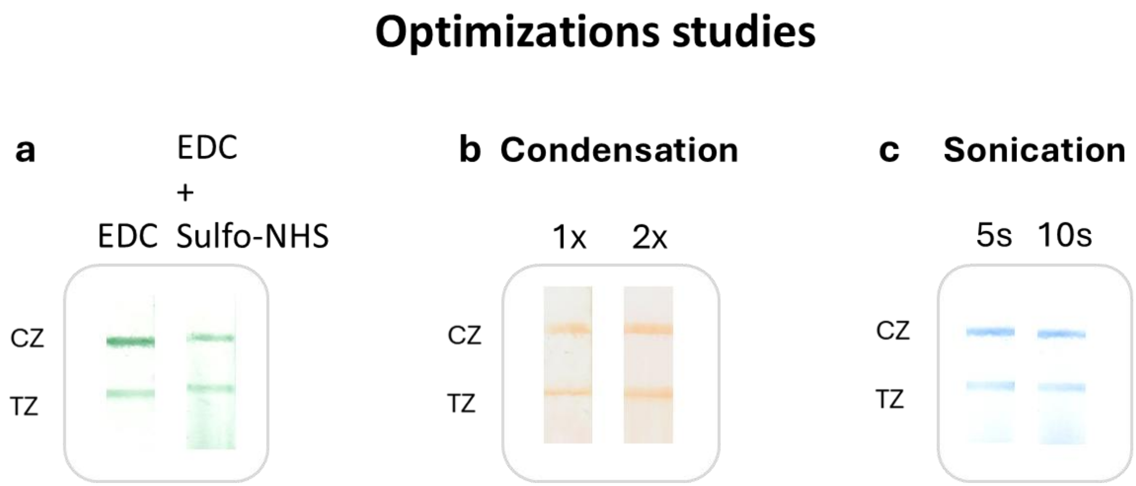

Fig. S7. Optimization studies of the coupling reaction on the beads. **a**. Use of EDC and EDC/sulfo-NHS. **b**. The effect of the condensation of the beads at half of the volume during the final resuspension of the conjugated beads. **c**. Sonication of the beads for 5 or 10 s between each step of the conjugation reaction. CZ: control zone, TZ: test zone.

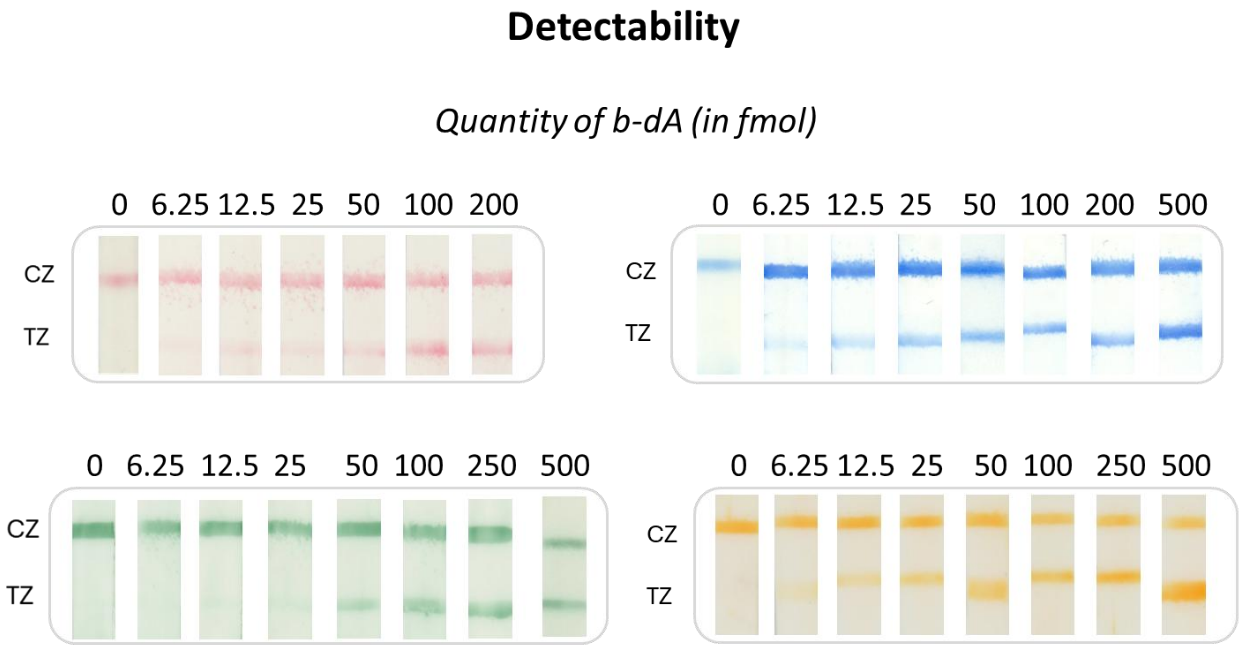

Fig. S8. Detectability of the rapid strip test for all four colored beads using different amounts of b-dA(30) (0-500 fmol) as target. CZ: control zone, TZ: test zone.

Real-time PCR

Real-time PCR was performed for SARS-CoV-2. The mixture contained 1×Kapa 2G Fast Ready Mix (Kapa Biosystems, Basel, Switzerland), 0.5 μM of the forward primer GACCCCAAAATCAGCGAAAT and 5 μM of the biotinylated reverse primer TCTGGTTACTGCCAGTTGAATCTG, 0.2 μL of 100×diluted SYBR-Green I (10000× concentrated) and 1 μL of the SARS-CoV-plasmid of various concentrations (0-10^4 DNA copies). The conditions of the amplification reaction were: a first step of denaturation at 95 °C for 3 min followed by 35 cycles of 95 °C for 5 s, 60 °C for 20 s and 72 °C for 5 s, and a final extension step at 72 °C for 1 min. The results are presented in Figure S9.

DNA copies for SARS-CoV-2	Cq
0	34.08
10	34.09
10^2	30.75
10^3	26.76
10^4	23.89

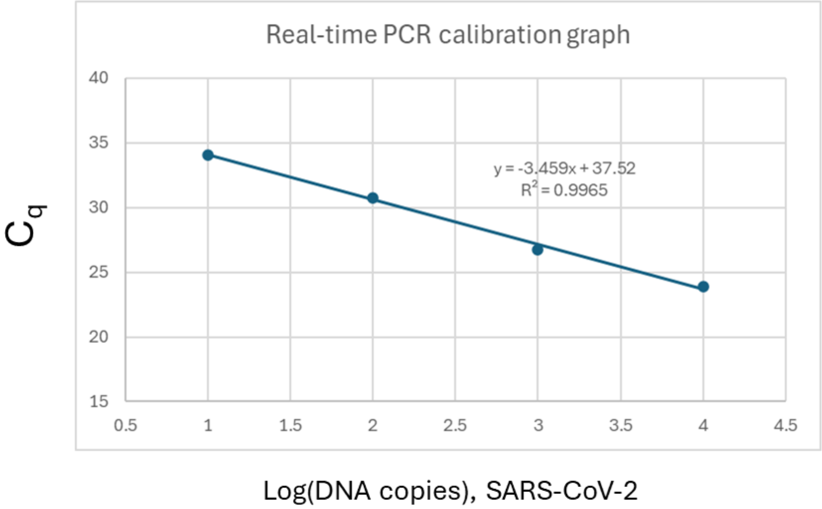

Fig. S9. Calibration graph for SARS-CoV-2 using real-time PCR. As low as 10^2 DNA copies of plasmid DNA for SARS-CoV-2 were detectable by real time PCR. Cq: threshold cycle.

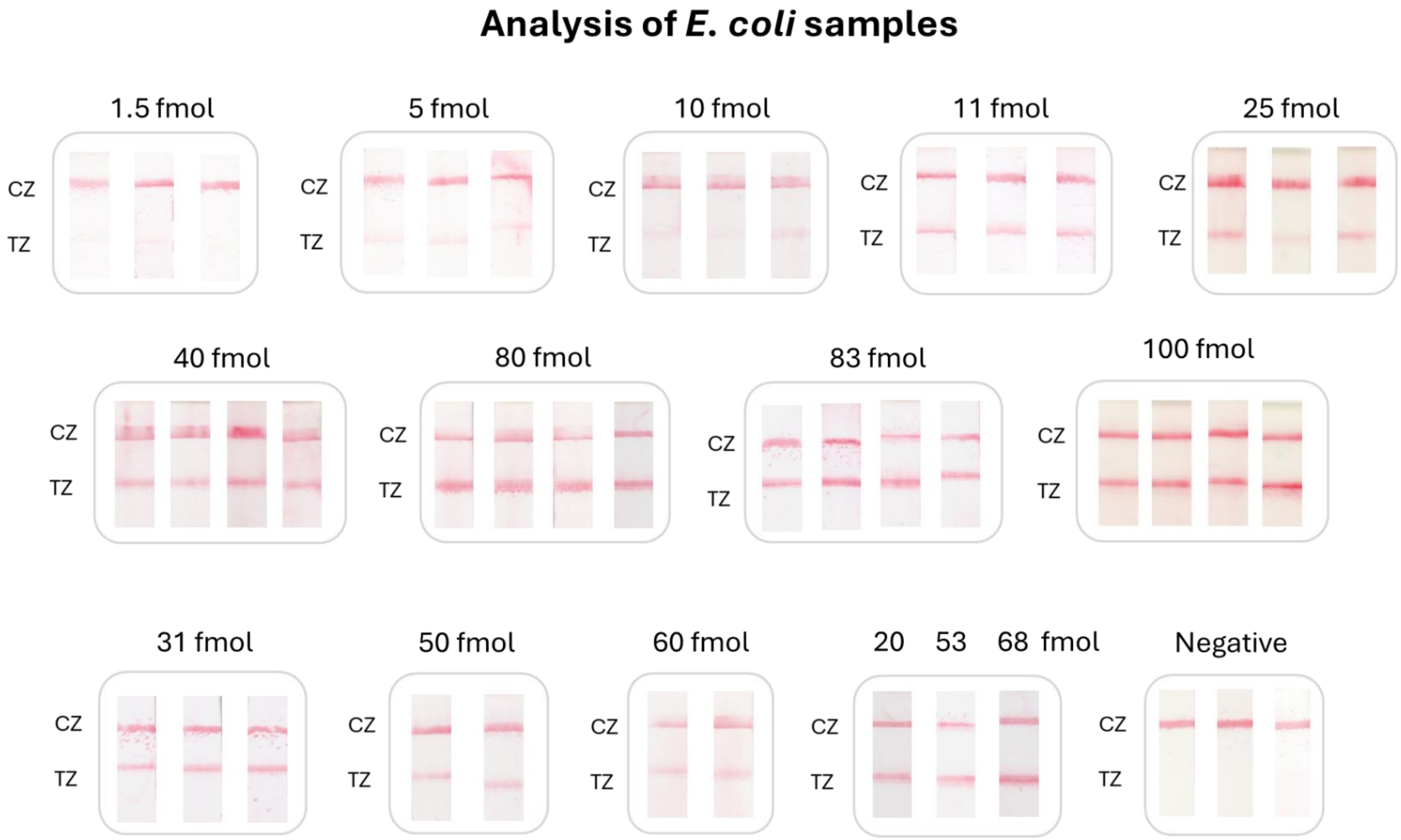

Fig. S10. Analysis of *E. coli* samples. Different prepared samples containing different amounts of the target were analyzed by the developed system. CZ: control zone, TZ: test zone.

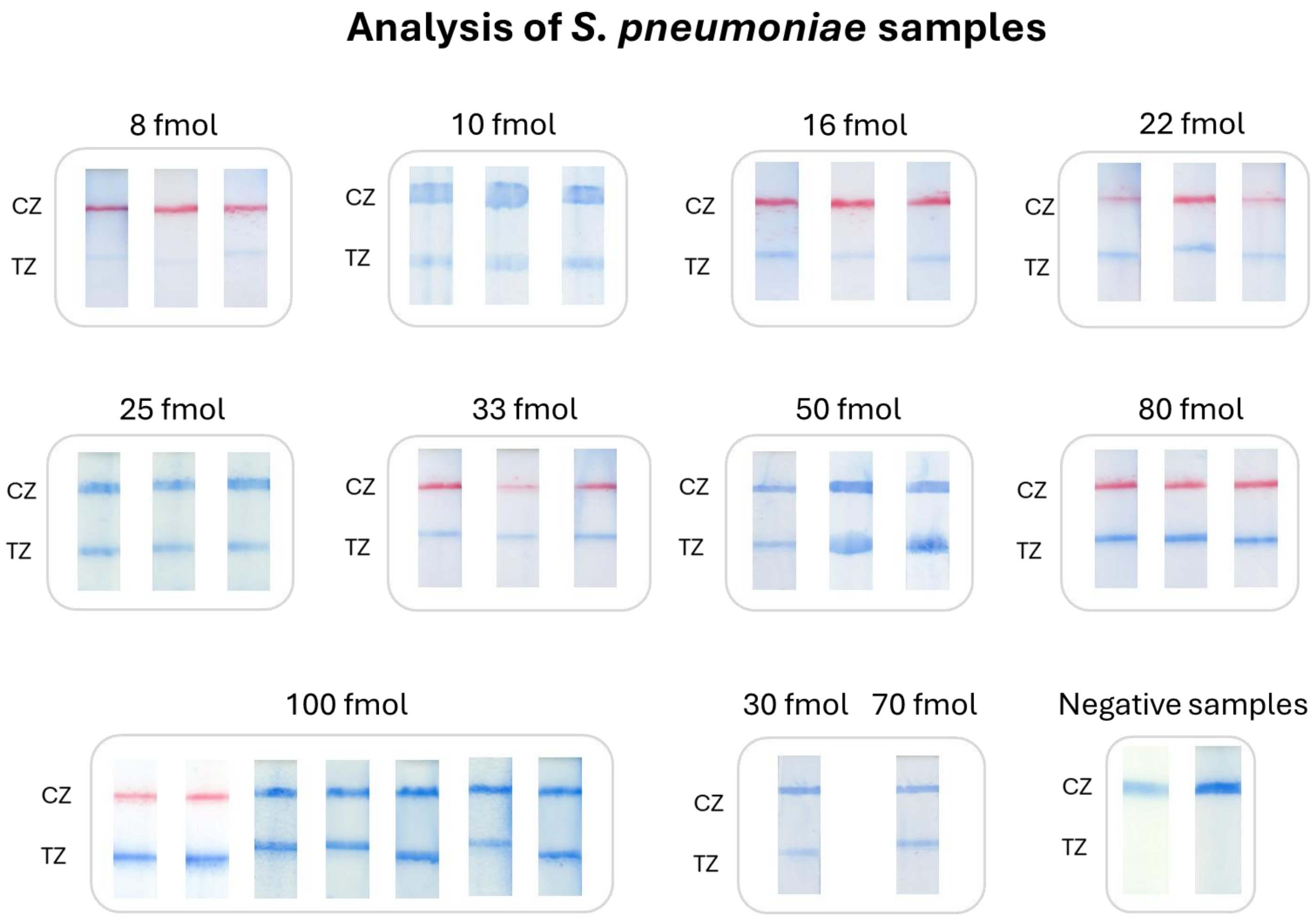

Fig. S11. Analysis of *S. pneumoniae* samples. Different prepared samples containing different amounts of the target were analyzed by the developed system. CZ: control zone, TZ: test zone.

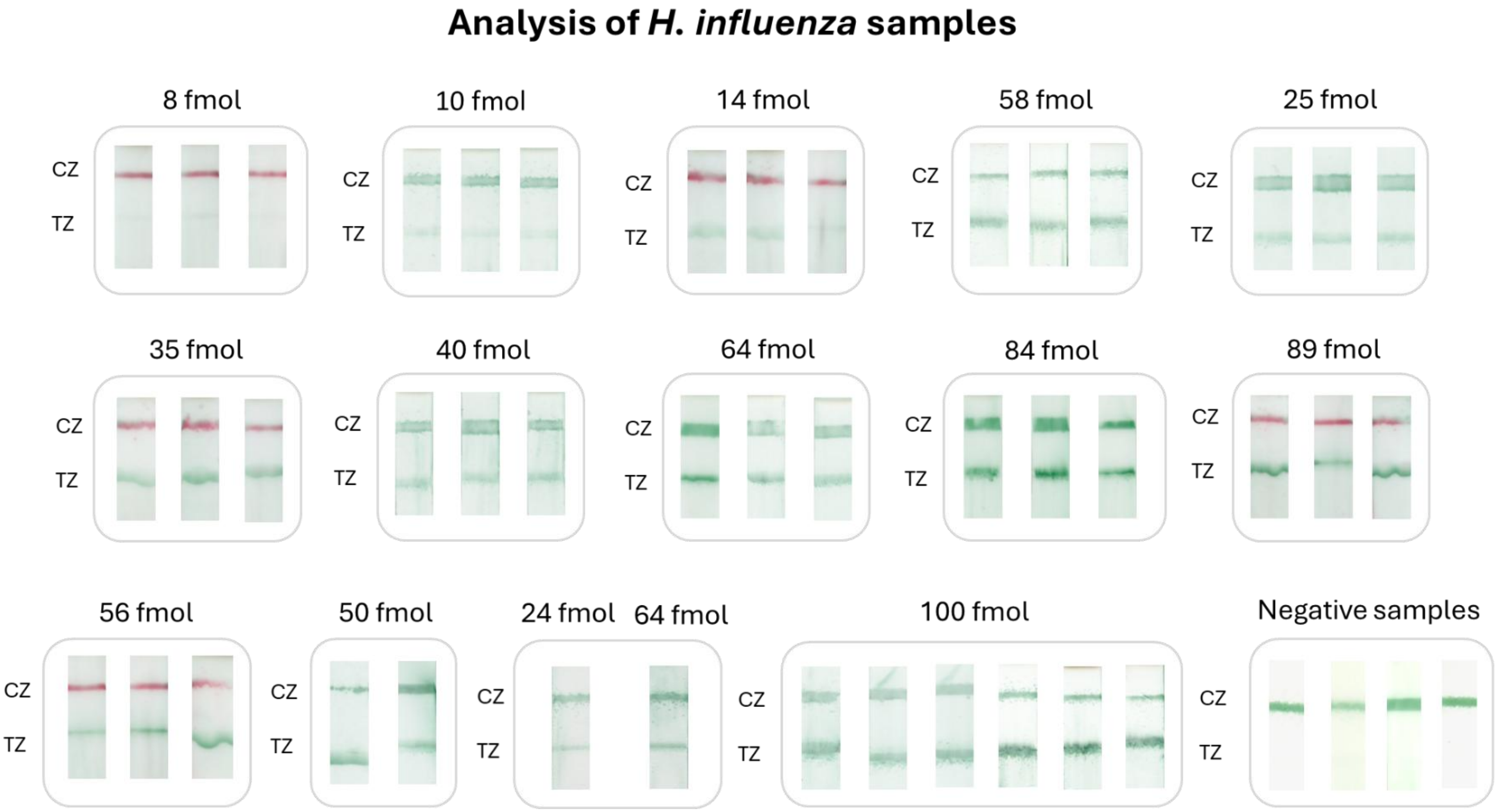

Fig. S12. Analysis of *H. influenza* samples. Different prepared samples containing different amounts of the target were analyzed by the developed system. CZ: control zone, TZ: test zone.

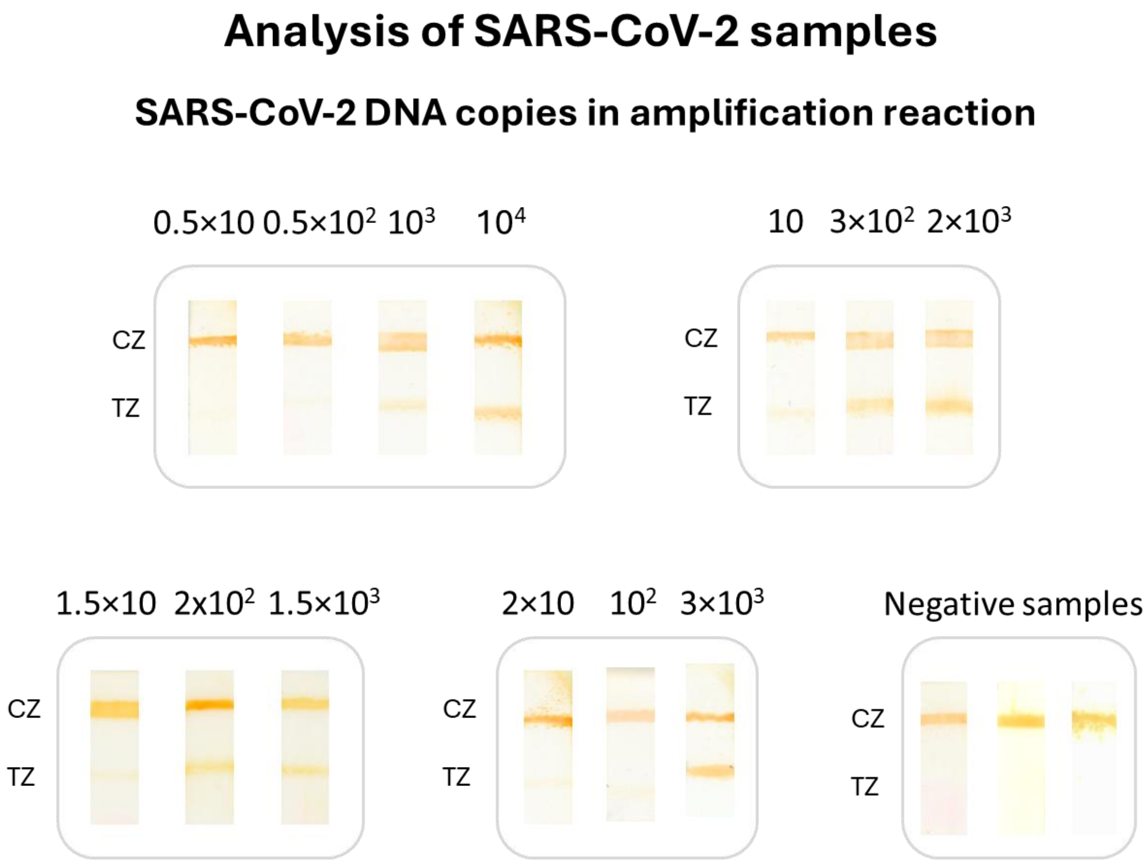

Fig. S13. Analysis of SARS-CoV-2 samples. Different samples containing different amounts of the target were analyzed by the developed system. CZ: control zone, TZ: test zone.

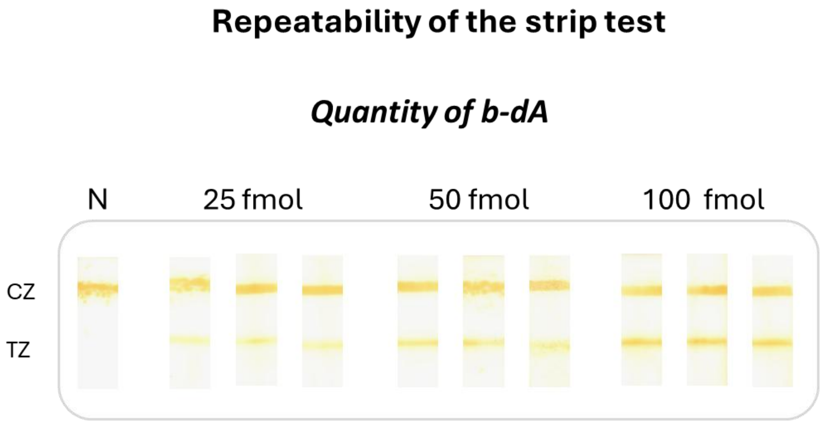

Fig. S14. Repeatability of the multicolor rapid test. Different amounts of b-dA(30) probe (25, 50 and 100 fmol) were analyzed in triplicate by the strip test. CZ: control zone, TZ: test zone.

Analysis of SARS-CoV-2 real samples

Real-time PCR

SARS-CoV-2 samples	Cq
Negative (N)	-
S1	-
S2	-
S3	16.98
S4	20.51
S5	21.92
S6	36.98
S7	-
S8	-
S9	21.53
S10	26.36
S11	27.48

Multicolor rapid test

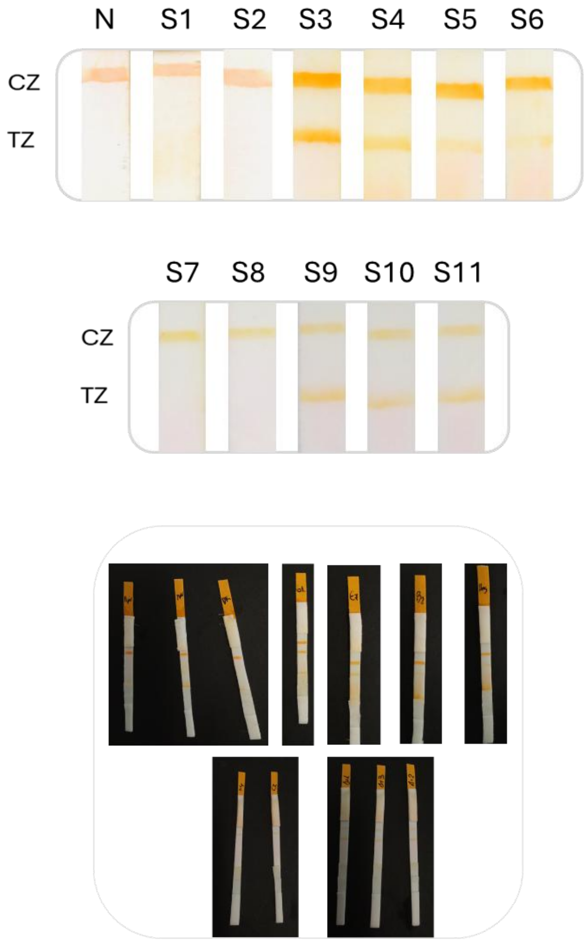

Fig. S15. Analysis of real samples for SARS-CoV-2 by real-time PCR and the multicolor rapid test - lateral flow assay. Cq: threshold cycle, CZ: control zone, TZ: test zone.

Table S2 Repeatability of image analysis-based application. The results of the analysis of samples in triplicates and the color identified by the application are reported. Images were captured by the smartphone Xiaomi Redmi Note 10.

Target, amount (fmol)	Result (Color identified) in triplicates
E. coli , 10 fmol	Red, Red, Red
E. coli , 31 fmol	Red, Red, Red
E. coli , 40 fmol	Red, Red, Red
E. coli , 80 fmol	Red, Red, Red
E. coli , 83 fmol	Red, Red, Red
E. coli , 20,53,68 fmol	Red, Red, Red
H. influenza , 10 fmol	Shamrock Green, Shamrock Green, Shamrock Green
H. influenza , 25 fmol	Shamrock Green, Shamrock Green, Shamrock Green
H. influenza , 40 fmol	Shamrock Green, Shamrock Green, Shamrock Green
H. influenza , 58 fmol	Shamrock Green, Shamrock Green, Shamrock Green
H. influenza , 84 fmol	Shamrock Green, Shamrock Green, Shamrock Green
H. influenza , 100 fmol	Shamrock Green, Shamrock Green, Shamrock Green
Streptococcus , 10 fmol	Cabo Blue, Cabo Blue, Cabo Blue
Streptococcus , 40 fmol	Cabo Blue, Cabo Blue, Cabo Blue
SARS-CoV-2 A	Orange, Orange, Orange
SARS-CoV-2 B	Orange, Orange, !Orange
SARS 10^2 molecules	Orange, Orange, Orange
Mixture of *Streptococcus*- blue 44 fmol - *E. coli*- red 58 fmol	Red, Cabo Blue, Red, Cabo Blue, Red, Cabo Blue
Mixture of *H. influenza* 64 fmol - *E. coli* 60 fmol	Red, Shamrock Green, Red, Shamrock Green, Red, Shamrock Green

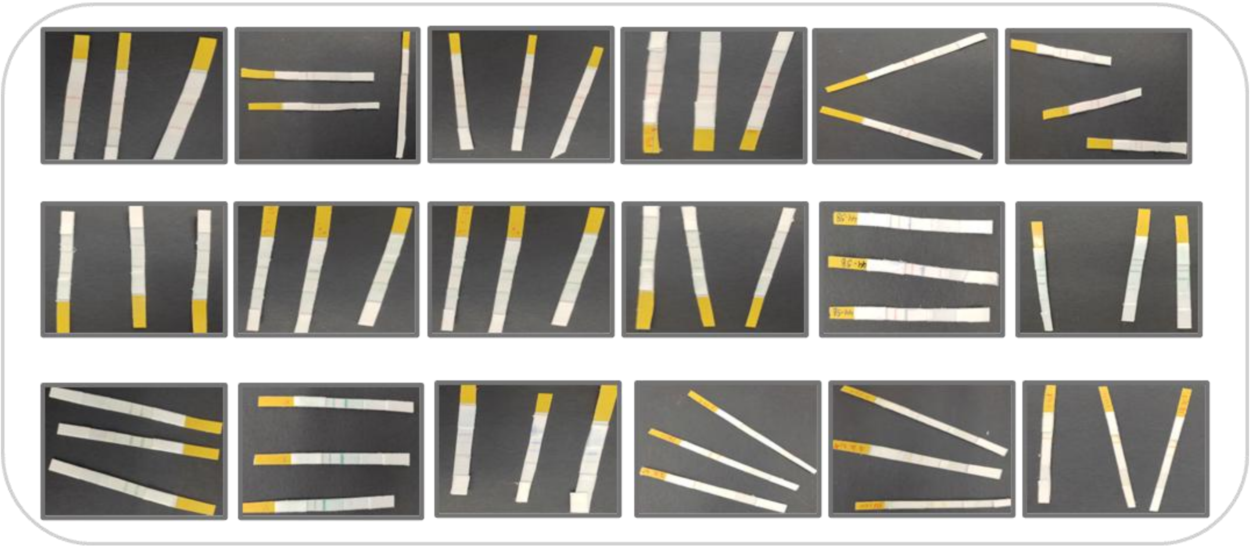

Fig. S16. Photos from the samples in triplicates used for application processing.

Table S3 Intra- and inter-repeatability of the AI and image analysis-based application.

Sample	Mobile device	True classification	Number of strips analyzed	Classification by the application
S. pneumoniae	Xiaomi Redmi note 11	Negative	n=9	Negative
S. pneumoniae	Huawei P20	Negative	n=5	Negative
S. pneumoniae, 100 fmol	Huawei P20	Positive	n=14	Positive
S. pneumoniae, 25 fmol	Huawei P20	Positive	n=4	Positive
S. pneumoniae and *E. coli*, 100 fmol	Huawei P20	Positive	n=5	Positive:4<br>Negative:1
S. pneumoniae and *E. coli*, 25 fmol	Huawei P20	Positive	n=7	Positive:5<br>Negative:2
E. coli and *H. influenza*, 100 fmol	Huawei P20	Positive	n=5	Positive
E. coli and *H. influenza*, 25 fmol	Huawei P20	Positive	n=8	Positive
S. pneumoniae and *H. influenza*, 100 fmol	Huawei P20	Positive	n=13	Positive
SARS positive sample 1	Huawei P20	Positive	n=6	Positive
SARS positive sample 2	Huawei P20	Positive	n=7	Positive:6<br>Negative:1
SARS positive sample 2	Lenovo Tablet 10	Positive	n=3	Positive
SARS positive sample 3	Huawei P20	Positive	n=16	Positive:14<br>Negative:2
SARS positive sample 3	Xiaomi Redmi note 11	Positive	n=3	Positive
SARS positive sample 4	Lenovo Tablet 10	Positive	n=3	Positive
SARS positive samples 5-7	Xiaomi Redmi note 11	Positive	n=3	Positive
SARS negative sample	Xiaomi Redmi note 11	Negative	n=3	Negative
SARS negative sample	Lenovo Tablet 10	Negative	n=3	Negative
